# Effects of Partial Fishmeal Replacement With Methanotrophic Bacterial Protein (*Methylococcus capsulatus*) in Siberian Sturgeon (*Acipenser baerii*) on Growth Performance, Nutrient Utilization, Body Composition, Immune Responses, Liver Histology, Gene Expression, and Gut Microbiota

**DOI:** 10.1155/anu/3788480

**Published:** 2026-11-02

**Authors:** Hien Van Doan, Pavel Hyrsl, Eliska Zuskova, Roey Angel, Ales Tomcala, Tram Thi Nguyen, Mahyar Zare, Ghasem Rashidian, Vlastimil Stejskal, Hung Quang Tran

**Affiliations:** ^1^ Department of Animal and Aquatic Sciences Faculty of Agriculture Chiang Mai University Chiang Mai 50200 Thailand cmu.ac.th; ^2^ Department of Experimental Biology Faculty of Science Masaryk University Kamenice 5 62500 Brno Czech Republic muni.cz; ^3^ Faculty of Fisheries and Protection of Waters South Bohemian Research Center of Aquaculture and Biodiversity of Hydrocenoses Institute of Aquaculture and Protection of Waters University of South Bohemia in České Budějovice Na Sádkách 1780 370 05 České Budějovice Czech Republic jcu.cz; ^4^ Institute of Soil Biology and Biogeochemistry Biology Centre CAS 37005 Ceske Budejovice Czech Republic cas.cz

**Keywords:** growth, health, *Methylococcus capsulatus*, microbiota, nutrition, Siberian sturgeon

## Abstract

This study evaluated the effects of dietary methanotrophic bacterial protein from *Methylococcus capsulatus* (MCP) as a partial replacement for fishmeal in juvenile Siberian sturgeon (*Acipenser baerii*). Four isonitrogenous, isolipidic, and isoenergetic diets were formulated containing 0%, 5.25%, 10.50%, or 15.75% MCP, corresponding to 0%, 25%, 50%, and 75% replacement of fishmeal, respectively. A total of 540 fish (23.15 ± 0.35 g) were randomly assigned to experimental diets in triplicate groups of 45 fish per tank over 70 days. Dietary MCP did not significantly affect growth performance, organo‐somatic indices, or serum biochemical and hematological parameters. However, MCP inclusion significantly affected the apparent digestibility of dry matter, protein, ash, phosphorus, and several amino acids, as well as immune‐related responses and hepatic lipid metabolism. Complement component 3 and lysozyme activity decreased with increasing MCP inclusion, whereas the expression of selected immune‐related genes in the head kidney and hepatic cholesterol and cholesteryl ester concentrations were altered. The intestinal microbial community remained dominated by Fusobacteriota, Firmicutes, and Proteobacteria, with no significant differences in overall community structure among dietary treatments. Overall, MCP at 5.75% supported biological responses comparable to the control diet, whereas higher inclusion levels were associated with reduced nutrient utilization and alterations in immune‐related and hepatic metabolic responses. These findings indicate that MCP can serve as a partial fishmeal substitute in Siberian sturgeon diets, although its inclusion level should be carefully considered.

## 1. Introduction

The Siberian sturgeon (*Acipenser baerii*) is among the most widely farmed sturgeon species globally due to its adaptability across diverse aquaculture systems, fast growth rate, and the high economic value of its caviar and meat [1]. Over recent decades, its cultivation has expanded markedly across Europe, Asia, and North America, making a substantial contribution to the global sturgeon aquaculture sector, with total production reaching ~318,000 tonnes in 2023 [1, 2]. Feed plays a critical role in the success of Siberian sturgeon farming, accounting for ~38%–42.3% of total production costs [3]. High‐quality aquafeed for sturgeon supports optimal growth and health and may shorten the reproductive cycle [4]. In practice, diets for Siberian sturgeon typically consist of high‐protein feeds (~40%) formulated for salmonids or specialized for sturgeon [4], with primary protein sources including soybean meal, fishmeal, sorghum meal, and corn gluten meal [5]. Global aquafeed production reached an estimated 53 million metric tonnes in 2023 [6], and projected industry growth may strain the availability of conventional feed ingredients. Notably, global fishmeal and fish oil production has remained relatively stable over the past decade, at ~5 million and 1 million tonnes per year, respectively, despite increasing demand [7]. To address this challenge, numerous studies have explored alternative protein sources, such as rendered animal byproducts, oilseed meals, and microalgae, as partial replacements for fishmeal in aquafeeds for Siberian sturgeon, with promising results [3, 8–12]. However, global crop production remains vulnerable to climate change, extreme weather events, geopolitical conflicts, and economic instability, which collectively threaten the security of the aquafeed ingredient supply chain [13–16]. Therefore, identifying cost‐effective, sustainable, and protein‐rich feed ingredients and valorizing underutilized by‐products is essential to diversify the aquafeed ingredient base and ensure the long‐term sustainability of aquaculture [15].

*Methylococcus capsulatus* (MCP), a methanotrophic bacterium, has emerged as a promising alternative protein source for aquafeeds over the coming decade. By utilizing methane as both a carbon and energy source, *M. capsulatus* produces high‐protein biomass with a balanced amino acid profile and favorable nutritional properties. The resulting bacterial protein meal (MCP) offers substantial environmental advantages, including low greenhouse gas emissions, minimal land and freshwater requirements, and the potential for large‐scale production (projected at ~2 million tonnes in 10 years), with an estimated commercial cost of ~USD 1500 per tonne [15, 17–19].

Commercially developed products based on *M. capsulatus* (e.g., UniBio’s Uniprotein or Calysta’s FeedKind) have shown promising outcomes in aquafeeds for various fish species. Studies report improvements in growth performance, immune function, gut microbiota composition, immune‐related gene expression, blood biochemistry, digestibility, and antioxidative capacity. For instance, rainbow trout fed MCP–based diets showed enhanced resistance to furunculosis, supporting its safety and functionality as a feed ingredient [20]. Chen et al. [21] demonstrated that MCP could replace up to 45% of fishmeal in white shrimp (*Litopenaeus vannamei*) diets without compromising growth, while also improving hepatopancreatic oxidative status, gut microbiota structure, and disease resistance. Likewise, MCP supplementation significantly improved growth and intestinal microbiota composition in spotted seabass (*Lateolabrax maculatus*) [19], and did not adversely affect production performance or plasma biochemistry in Japanese yellowtail (*Seriola quinqueradiata*) at a 20% inclusion level [17]. Additionally, dietary MCP has been shown to upregulate pro‐inflammatory cytokines via *tlr2* gene activation in largemouth bass (*Micropterus salmoides*), with inclusion at 8.6% yielding beneficial effects on intestinal health compared to control diets [22]. These studies have demonstrated that MCP can partially replace fishmeal without necessarily compromising growth, but they have also reported species‐ and inclusion‐level‐dependent effects on digestibility, immune responses, antioxidant status, and intestinal health. Thus, the current evidence supports MCP as a promising protein ingredient but does not indicate that its nutritional value and physiological consequences are uniform across fish species.

Importantly, most previous studies have focused primarily on production performance and selected health indicators, whereas the relationship among MCP inclusion, nutrient utilization, and downstream physiological responses remains insufficiently characterized. In particular, it is unclear whether maintaining growth performance with increasing MCP replacement is accompanied by changes in the digestibility of individual nutrients and amino acids, innate immune function, hepatic lipid metabolism, or the structure of the intestinal microbial community. This distinction is important because unchanged growth does not necessarily indicate equivalent nutrient utilization or physiological responses to alternative protein sources (e.g., [23, 24]). A more integrated assessment is therefore needed to determine whether MCP replacement produces coordinated or divergent responses across nutritional, metabolic, immune, and microbial endpoints. Siberian sturgeon provides a relevant model for examining these responses because of its high‐protein dietary requirements [4] and its established use of alternative protein sources in aquafeeds. Although several alternative ingredients have been investigated in this species (e.g., [11, 25, 26]), the nutritional and physiological consequences of graded replacement of fishmeal with methanotrophic bacterial protein have not been comprehensively characterized. Therefore, the knowledge gap addressed in the present study is not simply whether MCP can support growth in Siberian sturgeon, but how increasing MCP inclusion influences nutrient utilization and whether alterations in immune‐related responses, hepatic lipid metabolism, and intestinal microbial ecology accompany changes in nutrient availability.

The present study evaluated graded MCP inclusion as a partial replacement for fishmeal in juvenile Siberian sturgeon using an integrated set of nutritional and physiological indicators. We assessed growth and feed utilization, apparent digestibility of major nutrients and amino acids, serum and innate immune parameters, hepatic lipid classes, histological characteristics, selected gene expression responses, whole‐body composition, and intestinal microbiota. This integrated approach was used to determine whether growth performance is maintained despite changes in nutrient utilization and physiological regulation, and to identify an inclusion level that provides an appropriate balance between fishmeal replacement and biological responses. Additionally, multicriteria decision analysis was employed to determine the optimal inclusion level of bacterial protein for Siberian sturgeon.

## 2. Materials and Methods

### 2.1. Animal Ethics Statement

All animal care and experimental procedures were conducted in accordance with the Czech national legislation and the European Union Directive 2010/63/EU on the protection of animals used for scientific purposes (ethical approval protocol no. MSMT‐6744/2018‐2).

### 2.2. Diet Formulation and Preparation

The methane‐utilizing bacterium *M. capsulatus* protein (MCP) was sourced from a commercial supplier (Uniprotein, Unibio, Roskilde, Denmark). Four experimental diets were formulated to be isonitrogenous (crude protein: 54% on a dry matter basis), isolipidic (ether extract: 10%), and isoenergetic (gross energy: 21 MJ/kg). In these diets, MCP inclusion was 5.25%, 10.50%, and 15.75% at the expense of fishmeal at 25%, 50%, and 75% (abbreviation of diet UNI25, UNI50, and UNI75, respectively; Table 1). All diets were manufactured as sinking pellets (~2 mm in diameter) using a commercial twin‐screw extruder. The proximate composition, essential amino acid profile, lipid classes, and fatty acid composition of the MCP and the experimental diets are presented in Table 1, Supporting Information 1: Tables S1 and S2.

**Table 1:** Formulation, proximate composition, and amino acid (% as it) of experimental diets.

Ingredients (% as it)	Ingredients		Experimental diets			
	FM	MCP	UNI0	UNI25	UNI50	UNI75
Fishmeal			21.00	15.75	10.50	5.25
MCP			0.00	5.25	10.50	15.75
Pea protein concentrate			21.00	21.00	21.00	21.00
Soybean meal			10.00	10.00	10.00	10.00
Wheat flour			20.50	19.56	18.50	17.57
Wheat gluten meal			18.80	18.90	19.10	19.20
Dicalcium phosphate			0.00	0.75	1.55	2.30
Lysine HCL			0.00	0.07	0.12	0.18
Methionine			0.00	0.02	0.03	0.05
Rapeseed oil			5.00	3.70	2.50	1.20
Fish oil			3.00	4.30	5.50	6.80
Premix^a^			0.50	0.50	0.50	0.50
Yttrium oxide (Y_2_O_3_)			0.20	0.20	0.20	0.20
Proximate composition (% dry matter)						
Dry matter	96.5	95.6	97.3	97.3	96.1	97.6
Crude protein	70.2	65.7	54.3	54.2	54.1	53.6
Crude lipid	7.8	8.6	9.9	10.5	10.4	10.2
Crude fiber	1.5	0.2	3.1	3.1	3.3	2.8
Ash	12.5	5.7	6.5	6.4	6.4	8.0
NFE	1.3	15.4	25.5	25.3	25.0	26.2
Gross energy (Mj/kg)^b^	20.1	21.5	21.3	21.4	21.3	21.0
Amino acids (% as it)						
Methionine	2.08	1.95	0.93	0.92	0.97	0.99
Threonine	2.82	3.12	1.75	1.96	1.95	1.91
Valine	3.31	3.12	1.71	2.30	2.11	2.14
Leucine + isoleucine	7.63	9.12	4.84	6.62	6.51	6.68
Phenylalanine	2.50	3.39	2.46	2.81	2.76	2.88
Histidine	1.34	1.33	1.08	1.08	1.03	1.10
Lysine	4.86	3.75	2.85	3.06	2.97	2.93
Arginine	3.72	3.87	2.98	3.18	3.07	2.93

*Note:* Nitrogen‐free extract (NFE) = Dry matter−(crude protein + crude lipid + ash + fiber).

Abbreviations: FM, fishmeal; MCP, methane‐utilizing bacteria (*Methylococcus capsulatus*) protein.

^a^Vitamin and mineral premix (per kilogram premix): vitamin A, 4,000,000 IU; vitamin D_3_, 300,000 IU; vitamin E, 30,000 mg; vitamin K_3_, 1000 mg; vitamin B_1_, 1500 mg; vitamin B_2_, 2000 mg; vitamin B_6_, 1200 mg; vitamin B_12_, 5 mg; biotin, 50 mg; vitamin B_9_, 500 mg; vitamin B_3_, 10,000 mg; vitamin B_5_, 5000 mg; choline chloride, 60,000 mg; vitamin C, 30,000 mg; iron (as FeSO_4_ · H_2_O), 4800 mg; manganese (as MnO), 6200 mg; and zinc (as ZnO), 6000 mg.

^b^Gross energy (MJ/kg) as gross energy content of protein (23.6 MJ/kg), lipid (39.5 MJ/kg), and NFE (17.2 MJ/kg).

### 2.3. Experimental Design

Juvenile Siberian sturgeons in good health were procured from Fischzucht Rhonforelle GmbH & Co. KG (Gersfeld, Rhon, Germany). Prior to the experiment, fish were acclimated for 2 weeks and fed a commercial diet (Alltech Coppens Supreme‐10, Alltech, the Netherlands).

Following acclimation, 540 fish (initial body weight: 23.15 ± 0.35 g) were randomly allocated to 12 rectangular tanks (300 L each), with three replicate tanks per dietary treatment and 45 fish per tank. The tanks were connected to a recirculating aquaculture system (RAS) consisting of four submerged biofilter units (3500 L each) filled with bio‐elements and four sump tanks of identical volume. Mechanical filtration was provided by filter foam and a drum filter (AEM ECO15, Kerkrade, the Netherlands).

Water inflow to each tank was maintained at 4–5 L/min using AquaForte pumps (Veghel, the Netherlands). Each tank was equipped with a sedimentation cone and a bottom valve for removing the uneaten feed and feces. Water temperature was controlled via an air‐conditioning system (Inverter, LG, Seoul, South Korea). A 12 h light:12 h dark photoperiod was maintained (07:00–19:00), with light intensity above the tanks set at 450 lx, measured using a digital luxmeter (DT‐8809A, Nanshan, Shenzhen, China).

Water quality parameters, including temperature, pH, and dissolved oxygen, were monitored daily using a multiparameter probe (HI98194, Hanna Instruments, Woonsocket, RI, USA). Ammonium (N–NH_4_^+^), ammonia (NH_3_), and nitrite (N–NO_2_^−^) concentrations were measured three times weekly using LCK cuvette tests with barcode identification and a spectrophotometer (DR 2800, Hach Lange, Loveland, CO, USA). Throughout the experimental period, mean water quality parameters were maintained as follows: temperature, 20.18 ± 0.67°C; oxygen saturation, 86.0 ± 2.8%; pH, 7.21 ± 0.34; N–NH_4_^+^, 0.33 ± 0.14 mg/L; NH_3_, 0.003 ± 0.001 mg/L; and N–NO_2_^−^, 0.03 ± 0.02 mg/L.

Fish were fed *ad libitum* twice a day (09:00 and 15:00) for 70 days. Approximately 30 min post‐feeding, tanks were partially drained to remove uneaten pellets, which were then oven‐dried to a constant weight (Universal UN75, Memmert GmbH & Co. KG, Schwabach, Germany) to determine the actual feed intake. Mortality was recorded daily throughout the trial.

### 2.4. Sample Collection, Calculations, and Analysis

#### 2.4.1. Production Performance

At the conclusion of the feeding trial, fish were fasted for 24 h prior to sampling. All fish in each tank were then individually counted and weighed to evaluate growth performance. Measurements were conducted under light anesthesia using eugenol (0.03 mL/L; Sigma–Aldrich, Missouri, USA) to minimize handling stress. Growth indices, survival rate, and feed efficiency were calculated as follows:

Survival rate (%) = 100 × The final number of fish/The initial number of fish; Daily weight gain (WG) (g/fish/day) = (*W*_*f*_ − *W*_*i*_)/*t*, where *W*_*f*_ is the final wet weight, *W*_*i*_ is the initial wet weight, and *t* is the number of days; Specific growth rate (SGR,  %/day) = [(ln *W*_*f*_ − ln *W*_*i*_)/*t*] × 100; Feed conversion ratio (FCR) = total feed fed (g)/WG (g); Protein efficiency ratio (PER) = WG (g)/dry protein intake (g); Feed intake (g/fish/day) = total feed (g)/total fish/70.

#### 2.4.2. Nutrient Digestibility

Feces were collected from each tank starting on day 21 of the experiment to determine the apparent nutrient digestibility of the experimental diets. Samples were collected after the final daily feeding (15:00 h), following the removal of the uneaten feed, by gently siphoning from the tank bottom. The collected feces were immediately stored at −20°C until further analysis. The apparent digestibility coefficients (ADC, %) of dry matter, crude protein, lipid, ash, phosphorus, and amino acids were calculated according to the method described by Cho and Slinger [27], using the following equation:

ADC %=100−100×%Y2O3 in diet/%Y2O3 in feces×%Nutrient in feces/%Nutrient in diet.

#### 2.4.3. Hematology and Biochemistry

At the end of the feeding trial, four fish per tank (*n* = 12 fish per treatment) were randomly sampled following 24 h of feed deprivation and euthanized by an overdose of eugenol (0.3 mL/L water). Blood samples (~1.5 mL per fish) were collected from the caudal vein and divided into two aliquots. One aliquot, treated with heparin, was used for hematological analysis. The second aliquot, without anticoagulant, was centrifuged at 3000 ×*g* for 10 min at 4°C. The resulting serum was separated and stored at −80°C until further analyses. Hematocrit was determined after centrifugation of heparinized blood at 14,000 rpm for 15 min at 4°C using a Heraeus Megafuge 16 R centrifuge. Red blood cells (RBCs) and white blood cells (WBCs) were counted using a Neubauer hemocytometer, as previously described by Zare et al. [28].

Serum biochemical parameters, including triglycerides, aspartate aminotransferase, cholesterol, total protein, glucose, and albumin, were analyzed using an automatic analyzer (Architect c4000, Abbott Laboratories, IL, USA) according to the manufacturer’s instructions. Total IgM levels were determined as described by Rohlenová et al. [29]. Complement components (C3 and C4) and lysozyme activity were measured using commercial assay kits (MyBioSource, CA, USA).

#### 2.4.4. Somatic Indices

The liver, intestine, and viscera were carefully excised immediately after sampling and weighed to determine the organosomatic indices. These indices were calculated using the following formulas: hepatic somatic index (HSI, %) = 100 × liver weight (g)/body weight (g); visceral somatic index (VSI, %) = 100 × viscera weight (g)/body weight (g); and gastrointestinal somatic index (GSI, %) = 100 × intestine weight (g)/body weight (g).

#### 2.4.5. Liver Histology

Immediately after blood sampling, nine fish per treatment group were euthanized by an overdose of eugenol (0.3 mL/L water), and liver samples were collected and fixed in 10% buffered formalin for histological analysis. After 24 h of fixation, the tissues were processed using standard histological procedures. Samples were dehydrated with a tissue processor (Histomaster 2052/1.5, MDS Group, Germany), embedded in paraffin, sectioned using a rotary microtome, and stained with hematoxylin and eosin (H&E) using an automatic slide stainer (Tissue‐Tek DRS 2000, Sakura, USA). Histological sections were examined under a light microscope (Olympus BX51, Japan) equipped with a digital camera system. Histological alterations were semi‐quantitatively scored as absent (0), mild (1), moderate (2), or severe (3). The histological assessment was performed in a blinded manner to dietary treatment. The evaluated histopathological parameters included hyperemia, intracellular edema, and steatosis.

#### 2.4.6. Gene Expression in Liver, Head Kidney, and Intestine

Nine fish per dietary treatment (three fish from each replicate tank) were randomly selected for analysis of relative gene expression. After anesthetizing the fish with an overdose of eugenol (0.3 mL/L water), 0.5 g of tissue samples from the head, kidney, liver, and intestine were collected and immediately preserved in RNAlater (Thermo Fisher Scientific, USA). Total RNA was extracted using TRIzol reagent (Invitrogen, USA) and the PureLink RNA Mini Kit (Invitrogen), according to the manufacturer’s instructions. The quality and quantity of the extracted RNA were assessed using a NanoDrop One spectrophotometer by measuring the absorbance ratios at 260/280 and 260/230 nm, respectively. Subsequently, 1 μg of total RNA was used to synthesize cDNA with iScript Reverse Transcription Supermix (Bio‐Rad, USA) for quantitative reverse transcription PCR (qRT‐PCR). The qRT‐PCR reaction mixture consisted of 1 μL of cDNA (100 ng), 1 μL of each primer (4 μM), 5 μL of 2 × iTaq Universal SYBR Green Supermix (Bio‐Rad), and distilled water to a final volume of 10 μL. The qRT‐PCR was performed using a CFX Connect Real‐Time System (Bio‐Rad). The amplification protocol consisted of an initial denaturation at 95°C for 30 s, followed by 40 cycles of denaturation at 95°C for 15 s and annealing at 60°C for 30 s. This was followed by a melt‐curve analysis at 95°C for 15 s, 60°C for 60 s, and 95°C for 15 s. Each cDNA sample was analyzed in duplicate for each target gene and reference gene. The qRT‐PCR results were evaluated using the 2^−ΔΔCt^ method, and a standard curve was plotted [30]. The full protocol was previously described by Le Xuan et al. [31]. The primer sequences used for gene amplification are listed in Supporting Information 1: Tables S4–S6.

#### 2.4.7. Lipid, Fatty Acids, and Lipid and Phospholipid Classes

The remaining liver samples from Section 2.4.6 were stored at −20°C for subsequent lipid class analysis. Intact lipids in the total lipid extract were separated by thin‐layer chromatography (TLC) to determine the composition of individual lipid and phospholipid classes, according to Olsen and Henderson [32], with minor modifications. Briefly, samples were prepared in chloroform:methanol (2:1, v/v) at ~1 μg/mL. Aliquots were applied to TLC plates using a CAMAG ATS 4 automatic TLC sampler (CAMAG, Switzerland). Lipid classes were identified by comparison with external phospholipid and mixed lipid standards (TLC 18‐3A and TLC 18‐5C; Nu‐Check Prep, Elysian, MN, USA).

#### 2.4.8. Fish Gut Microbiota

On day 70 of the feeding trial, three fish per tank (*n* = 9 per diet) were randomly sampled and euthanized with an overdose of eugenol (0.3 mL/L). Posterior intestinal digesta were gently collected into sterile 1.5 mL tubes and stored at −80°C until analysis. DNA was extracted as previously described [33], using three consecutive bead‐beating cycles (Lysing Matrix E, MP Biomedicals) in a FastPrep‐24 5G instrument. DNA concentration was determined with the Quant‐iT PicoGreen assay (Thermo Fisher Scientific). The V4 region of the 16S rRNA gene was amplified using primers 515F_mod and 806R [34] with Fluidigm CS1/CS2 linkers and sequenced in a two‐step protocol on an Illumina MiniSeq platform [35]. Sequencing was performed at the DNA Services Facility at the University of Illinois, Chicago (USA). Raw reads were processed in Cutadapt v3.5 [36] to remove primers and linkers. Alpha diversity indices were calculated in the vegan package [37] and compared using the Kruskal–Wallis test followed by Dunn’s post hoc test [38] and Dunn’s test [39]. Beta diversity was assessed using constrained analysis of principal coordinates (CAP)–based on Bray–Curtis dissimilarities, and differences among diets were tested with PERMANOVA [40].

#### 2.4.9. Whole Body Composition

At the end of the feeding trial, following 24 h of feed deprivation, four fish per tank (12 fish/diet) were randomly collected and sacrificed by a sharp blow on the head. Fish samples were freeze‐dried in an oven (HervestRight, Utah, USA), ground into powder using a grinder (PureEase Food processor FP 3131 WH, Braun, Neu‐Isenburg, Germany), and stored at –20°C for further compositional analysis.

#### 2.4.10. Nutritional Composition Analysis

Ingredients, experimental diets, feces, and whole‐body fish samples were thoroughly homogenized and analyzed according to AOAC [41] procedures for dry matter (method 934.01), crude ash (method 942.05), and crude fiber (method 991.43). Crude protein (method 990.03) was determined using the Kjeldahl method with an automatic Kjeldahl system (Buchi, Flawil, Switzerland). Total lipid content (method 954.02) and fatty acid profiles were analyzed as described by Mráz and Pickova [42]. Phosphorus content was measured using an inductively coupled plasma atomic emission spectrophotometer (ICP‐OES; Prodigy7, Leeman Labs, USA). Yttrium oxide (Y_2_O_3_) concentrations in diets and fecal samples were determined by ICP‐OES after nitric acid digestion at 180°C for 48 h. The amino acid profiles of the ingredients and the experimental diets were analyzed as described by Opekar et al. [43].

#### 2.4.11. Statistical Analyses

All statistical analyses were performed in the R statistical package (RStudio 2025.05.0 Build 496, 2009–2025 Posit Software, PBC). The data obtained were assessed for normality (Shapiro–Wilk’s test) and homogeneity of variances (Levene’s test). The tank was considered the experimental unit for dietary comparisons. When multiple fish were sampled from the same tank, measurements were averaged within the tank before statistical analysis. One‐way ANOVA was used to test the differences in the tested indices across dietary groups. Tukey’s post‐hoc test was used when appropriate. Differences were regarded as significant at *p* < 0.05.

To provide an exploratory visualization of relationships among the measured biological responses, principal component analysis (PCA) was performed using dietary treatment‐level mean values. Individual fish measurements were not treated as independent observations in this analysis. Gene‐expression variables were excluded because they constituted a separate molecular‐response dataset with multiple target genes and different measurement scales. Variables measured on different scales were standardized as *z*‐scores before PCA. To minimize the influence of redundant information, pairwise correlations among variables were examined, and highly correlated variables representing closely related measurements were reduced before the final PCA. The PCA is implemented by the “*fviz_pca_biplot*” package. A heatmap was generated using the same selected variables after standardization, with hierarchical clustering applied to visualize similarities among dietary treatments and biological variables, and was plotted using the “*pheatmap*” package.

## 3. Results

### 3.1. Production Performance and Somatic Indices

Following a 70‐day feeding trial, fish showed no significant differences in final weight, daily weight gain, specific growth rate, feed intake, visceral somatic, and gastrointestinal index (*p* > 0.05; Table 2).

**Table 2:** Growth performance and somatic indices of Siberian sturgeon (*Acipenser baerii*) fed four experimental diets for 70 days.

Growth indices	Experimental diets				SEM	ANOVA *p*‐Value
	UNI0	UNI25	UNI50	UNI75		
Initial body weight (g/fish)	22.88	23.33	23.30	23.10	0.10	0.756
Final body weight (g/fish)	98.69	94.83	96.14	98.02	1.68	0.883
Daily weight gain (g/fish/day)	1.08	1.02	1.04	1.07	0.02	0.847
Specific growth rate (%/day)	2.09	2.00	2.02	2.06	0.03	0.706
Feed intake (g/fish/day)	0.74	0.72	0.73	0.74	0.01	0.259
Feed conversion ratio	0.88	0.95	1.01	1.05	0.02	0.084
Survival rate (%)	91.11	91.85	91.11	85.19	1.82	0.601
Visceral somatic index (%)	6.29	6.38	6.38	6.74	0.12	0.623
Hepatic somatic index (%)	1.63	1.71	1.72	1.95	0.05	0.080
Gastrointestinal somatic index (%)	4.02	3.83	3.79	4.03	0.08	0.678

Abbreviation: SEM, standard error of means.

### 3.2. Nutrient Digestibility

Dietary treatments significantly affected nutrient digestibility for dry matter (*p* = 0.011), protein (*p* < 0.001), lipid (*p* < 0.001), phenylalanine (*p* = 0.023), and arginine (*p* = 0.015), while other nutrients remained comparable across diets (Table 3). Fish fed MCP‐free diet digested significantly higher dry matter, protein, phenylalanine, and arginine than those fed the UNI75 (*p* < 0.05).

**Table 3:** Nutrient digestibility (%) of Siberian sturgeon fed four experimental diets for 70 days.

	Experimental diets				SEM	ANOVA *p*‐Value
	UNI0	UNI25	UNI50	UNI75		
Dry matter	78.89^a^	77.28^ab^	76.17^ab^	73.97^b^	0.63	0.011
Protein	93.68^a^	92.3^b^	91.1^bc^	89.89^c^	0.44	<0.001
Lipid	92.47^c^	93.87^b^	97.01^a^	97.67^a^	0.66	<0.001
Ash	68.05	63.05	60.98	65.10	1.01	0.049
Phosphorus	73.39	67.81	60.73	57.17	1.98	<0.001
Methionine	99.06	97.79	97.71	96.94	0.31	0.087
Threonine	94.04	92.89	91.64	89.93	0.65	0.115
Valine	92.37	93.04	92.85	88.32	0.80	0.094
Leucine + isoleucine	92.85	93.19	92.9	90.06	0.56	0.157
Phenylalanine	95.02^a^	93.95^ab^	93.33^ab^	91.66^b^	0.45	0.023
Histidine	93.13	92.66	92.83	91.26	0.55	0.698
Lysine	95.98	95.26	95.28	94.66	0.22	0.224
Arginine	96.9^a^	96.31^ab^	95.95^ab^	94.91^b^	0.26	0.015

*Note:* Different letters within a row indicate significant differences (*p* < 0.05).

Abbreviation: SEM, standard error of means.

### 3.3. Hematology and Serum Biochemistry

Dietary MCP did not significantly affect the measured serum biochemical or hematological parameters (*p* > 0.05). In contrast, complement C3 and lysozyme activity were significantly affected by dietary treatment (*p* = 0.024 for each; Table 4).

**Table 4:** Serum physiological and biochemical indices of Siberian sturgeon fed experimental diets for 70 days.

	Experimental diets				SEM	ANOVA *p*‐Value
	UNI0	UNI25	UNI50	UNI75		
Biochemistry						
Triglyceride (mmol/L)	2.00	1.51	1.76	1.65	0.16	0.771
AST (µkat/L)	15.83	16.75	15.16	12.64	0.88	0.384
Cholesterol mmol/I	1.80	1.29	1.46	1.48	0.07	0.072
Protein (g/L)	13.91	11.58	12.5	13.00	0.36	0.163
Glucose (mmol/L)	1.77	1.71	2.12	1.94	0.10	0.422
Hematology						
Hematocrit (%)	56.93	51.63	50.49	50.2	1.21	0.171
WBC (cell/mL)	1.6 × 10^7^	1.3 × 10^7^	1.8 × 10^7^	1.5 × 10^7^	7.4 × 10^5^	0.204
RBC (cell/mL)	4.5 × 10^9^	4.0 × 10^9^	5.2 × 10^9^	5.5 × 10^9^	3.1 × 10^8^	0.461
Immunity						
Albumin (g/L)	4.72	3.88	4.37	4.33	0.14	0.262
IgM (mg/mL)	14.47	15.07	15.25	13.83	0.78	0.923
C3 (µg/mL)	79.03^a^	68.85^ab^	68.36^ab^	60.63^b^	2.12	0.024
C4 (µg/mL)	4.98	5.3	4.88	5.04	0.24	0.941
Lysozyme (U/mL)	49.39^a^	41.34^ab^	43.9^ab^	36.58^b^	1.48	0.024

*Note:* Data are presented as the mean (*n* = 4 fish/tank or 12 fish/group), values in the same row with different superscripts are significantly different (*p* < 0.05).

Abbreviations: AST, aspartate aminotransferase; C3 and C4, complement C3 and C4; IgM, immunoglobulin M; RBC, red blood cell; SEM, standard error of the mean; WBC, white blood cell.

### 3.4. Lipid Classes in Fish Livers

A total of 10 lipid fractions, classified as polar and neutral lipids, were extracted from sturgeon livers (Table 5). No significant differences were observed in hepatic polar lipid fractions among fish fed the experimental diets (*p* > 0.05). In contrast, two neutral lipid fractions were significantly influenced by dietary treatments. Specifically, hepatic cholesterol levels were significantly higher in the UNI25 group compared to UNI75 (*p* = 0.009). Conversely, UNI50 and UNI75 groups exhibiting significantly higher hepatic cholesteryl ester concentrations than those in UNI25 (*p* = 0.004).

**Table 5:** Lipid classes (% total lipids) of livers of Siberian sturgeon fed experimental diets for 70 days.

Lipid classes	Experimental diets				SEM	ANOVA *p*‐Value
	UNI0	UNI25	UNI50	UNI75		
Sphingomyelin	1.04	0.91	0.69	0.74	0.09	0.539
Phosphatidylcholine	15.94	16.65	15.58	15.45	0.35	0.632
Phosphatidylinositol	5.47	5.10	4.84	5.57	0.30	0.825
Cardiolipin	4.75	4.43	5.02	6.01	0.37	0.475
Phosphatidylglycerol	4.03	3.54	3.33	3.85	0.22	0.693
Phosphatidylethanolamine	12.95	13.65	13.44	13.70	0.26	0.751
Total polar lipids	**44.18**	**44.29**	**42.90**	**45.32**	**44.18**	
Free fatty acid	6.42	6.91	5.85	6.05	0.20	0.255
Cholesterol	14.93^ab^	15.85^a^	14.68^ab^	13.86^b^	0.22	0.009
Triacylglycerol	30.01	32.96	29.87	27.52	1.01	0.309
Cholesteryl	4.47^ab^	0.00^b^	6.71^a^	7.25^a^	0.84	0.004
Total neutral lipids	**55.82**	**55.71**	**57.10**	**54.68**	**55.82**	

*Note:* Data are presented as the mean (*n* = 3 fish/tank or 9 fish/group), values in the same row with different superscripts are significantly different (*p* < 0.05).

Abbreviation: SEM, standard error of means.

### 3.5. Histology of Fish Liver

The structure of liver of Siberian sturgeon fed experimental diets is described in Supporting Information 2: Figure S1 and Supporting Information 1: Table S3. Histological examination revealed no significant differences among dietary treatments in the scores for hyperemia, intercellular edema, or steatosis (*p* > 0.05).

### 3.6. Gene Expression

The relative mRNA expression levels of genes related to energy metabolism, lipid metabolism, growth, and cellular stress responses in fish liver are shown in Figure 1. A significant downregulation of *pck1* expression was observed in the UNI25 and UNI75 groups compared to UNI0 (*p* < 0.05). In contrast, *hexok4* expression was significantly upregulated in UNI50 and UNI75 groups relative to both UNI0 and UNI25 (*p* < 0.05). The growth‐related gene *igf1* showed significantly higher expression in UNI25 than in UNI0 and UNI75 (*p* < 0.05), with the lowest level detected in UNI0. Notably, *gadd45*β, a gene involved in cellular stress and the cell cycle, was significantly upregulated in UNI75 compared to UNI25 (*p* < 0.05). No significant differences (*p* > 0.05) were detected in the expression levels of *fatp6*, *gck*, *fas*, *acc*, and *caspase* genes across the dietary treatments (Figure 1A).

**Figure 1:**
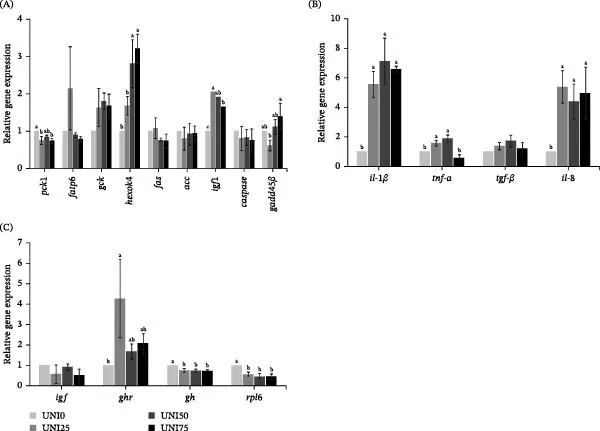
mRNA expression in liver (A), head kidney (B), and intestine (C) of Siberian sturgeon fed experimental diets for 70 days. Statistical analysis was performed via one‐way ANOVA. Different letters in the columns (a, b, c) indicate significant differences (*p* < 0.05).

Figure 1B presents the expression of immune‐related cytokine genes in the head kidney. The pro‐inflammatory cytokine *il-1*β was significantly upregulated in all MCP‐fed groups compared with UNI0 (*p* < 0.05). A similar pattern was observed for *tnf-α*, with significantly higher expression in UNI25 and UNI50 than in UNI0 (*p* < 0.05). Although the anti‐inflammatory cytokine *tgf-β* showed numerically higher expression in MCP‐fed groups, these differences were not statistically significant. The chemokine *il-8*, which is involved in neutrophil recruitment, was significantly upregulated in all MCP‐containing diets relative to the control (*p* < 0.05).

As shown in Figure 1C, *ghr* expression was significantly higher in fish fed the UNI25 diet compared with the control group (*p* < 0.05). In contrast, *igf* expression did not differ significantly among dietary treatments (*p* > 0.05). The expression of *gh* was significantly higher in UNI0 than in all MCP‐fed groups (*p* < 0.05), with no differences detected among UNI25, UNI50, and UNI75 (*p* > 0.05). A similar trend was observed for *rpl6*, which was significantly upregulated in UNI0 compared with all MCP treatments (*p* < 0.05), whereas no differences were observed among MCP‐fed groups (*p* > 0.05).

### 3.7. Whole Body Composition

Dietary inclusion of MCP significantly influenced whole‐body ash content and fatty acid composition (Table 6). Saturated and monounsaturated fatty acids in sturgeon were affected by dietary MCP (*p* < 0.001 and *p* = 0.006, respectively). In contrast, the proportions of health‐promoting fatty acids, including EPA and DHA, remained comparable among dietary treatments (*p* > 0.05).

**Table 6:** Whole body composition (% dry matter) and fatty acid profile (% total fatty acids) of Siberian sturgeon fed experimental diets for 70 days.

	Experimental diets				SEM	ANOVA *p*‐Value
	UNI0	UNI25	UNI50	UNI75		
Protein (%)	61.41	60.70	62.20	63.54	0.49	0.352
Lipid (%)	11.06	11.27	13.27	10.94	0.44	0.164
Ash (%)	11.96^b^	11.91^ab^	12.80^ab^	14.50^a^	0.27	0.004
Fatty acids						
C14:0	1.514^b^	1.472^ab^	1.453^ab^	1.411^a^	0.013	0.029
C14:1	0.061^d^	0.069^c^	0.077^b^	0.085^a^	0.002	<0.001
C16:0	14.835^d^	15.927^c^	16.560^b^	17.916^a^	0.179	<0.001
C16:1	1.869^d^	2.602^c^	3.416^b^	3.985^a^	0.127	<0.001
C18:0	3.03	3.133	3.021	3.471	0.089	0.238
C18:1n9 (OA)	40.338^a^	39.222^ab^	37.565^b^	35.948^bc^	0.323	<0.001
C18:1n7	3.623^a^	3.767^ab^	3.869^ab^	4.155^b^	0.047	<0.001
C18:2n6 (LA)	18.659	18.223	18.305	18.058	0.180	0.699
C18:3n3	2.618	2.532	2.628	2.415	0.045	0.308
C20:0	0.189^a^	0.177^a^	0.163^ab^	0.154^b^	0.003	<0.001
C20:1n9	3.714^a^	3.584^ab^	3.375^b^	3.248^b^	0.038	<0.001
C20:2n6	1.196	1.253	1.272	1.288	0.020	0.411
C20:4n6	1.096	1.101	1.184	1.25	0.035	0.354
C20:3n3	0.224^b^	0.236^ab^	0.258^a^	0.255^ab^	0.005	0.021
C22:0	0.013	0.008	0.003	0.003	0.002	0.421
C22:1n9	0.277^a^	0.270^ab^	0.256^ab^	0.249^b^	0.003	0.008
C20:5n3 (EPA)	1.243	1.201	1.302	1.23	0.022	0.413
C24:1n9	0.158^a^	0.145^ab^	0.135^b^	0.126^b^	0.003	<0.001
C22:5n6	0.562^b^	0.573^b^	0.642^a^	0.694^a^	0.011	<0.001
C22:6n3 (DHA)	4.782	4.506	4.515	4.058	0.120	0.198
SFA	19.581^c^	20.716^bc^	21.201^b^	22.955^a^	0.214	<0.001
MUFA	50.039^a^	49.66^a^	48.693^ab^	47.797^b^	0.258	0.006
PUFA	30.38	29.624	30.107	29.248	0.254	0.409
n‐3	8.867	8.475	8.703	7.958	0.136	0.092
n‐6	21.513	21.149	21.404	21.29	0.169	0.894
n‐3/n‐6	0.411	0.401	0.407	0.375	0.006	0.141
EPA + DHA	6.026	5.707	5.817	5.288	0.131	0.243

*Note:* Data are presented as the mean (*n* = 4 fish/tank or 12 fish/group), values in the same row with different superscripts are significantly different (*p* < 0.05).

Abbreviations: DHA, docosahexaenoic acid; EPA, eicosapentaenoic acid; LA, linoleic acid; MUFA, monounsaturated fatty acids; OA, oleic acid; PUFA, polyunsaturated fatty acids; SEM, standard error of the mean; SFA, saturated fatty acids.

### 3.8. Fish Gut Microbiota

Fusobacteriota, Firmicutes, and Proteobacteria were the predominant phyla in the intestinal microbiota of Siberian sturgeon, irrespective of dietary treatment (Figure 2A). No significant differences in phylum‐level relative abundance among dietary groups (*p* > 0.05). Significant differences in the relative abundances of Bacteroidota and Crenarchaeota between pre‐feeding and post‐feeding samples were found (*p* < 0.05; Supporting Information 1: Table S7). At the genus level, *Cetobacterium*, *Clostridium*, *Plesiomonas*, and *Aeromonas* were the most abundant taxa across all experimental groups (Figure 2B). Similarly, no significant differences were observed in the relative abundance of genera among the diets (*p* > 0.05). In contrast, comparison of the initial samples with those collected after the 70‐day feeding trial revealed a significant shift in bacterial community composition at the genus level (*p* < 0.05; Supporting Information 1: Table S8).

**Figure 2:**
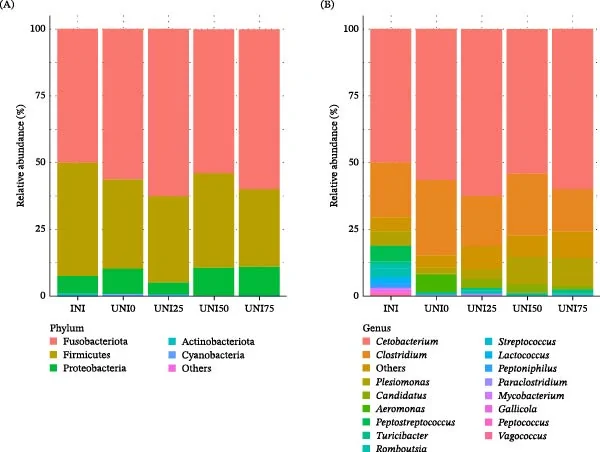
Stacked bar plot showing the bacteria’s relative abundance at the phylum (A) and genus (B) level in sturgeon. INI indicates a digesta sample of Siberian sturgeon at the start of the trial (*n* = 6); UNI0, UNI25, UNI50, and UNI75 represent experimental diets.

Alpha diversity indices did not differ significantly among dietary treatments. Specifically, the Shannon index (*p* = 0.073) and Simpson index (*p* = 0.064) showed no significant variation (Supporting Information 2: Figure S2). Principal coordinate analysis (PCoA)–based on Bray–Curtis dissimilarity was conducted using the relative abundance of ASVs with (Figure 3A) and without (Figure 3B) initial intestinal samples. PERMANOVA analysis confirmed that bacterial community structure did not differ significantly among dietary treatments (*p* = 0.300).

**Figure 3:**
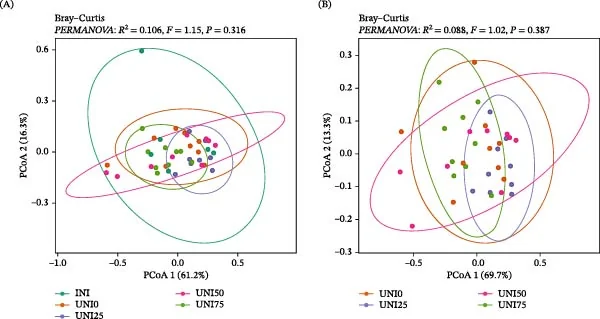
Principal coordinates analysis of intestine, with (A) and without (B) initial digesta samples (*n* = 9) based on the Bray–Curtis metric. INI indicates the digesta sample of Siberian sturgeon at the start of the trial (*n* = 6); UNI0, UNI25, UNI50, and UNI75 represent experimental diets.

### 3.9. Overall Effect of Experimental Diets on Siberian Sturgeon

The PCA biplot (Figure 4A) illustrates the relationships among experimental diets and their effects on various biological indices of Siberian sturgeon. UNI25 clusters are relatively close to UNI0, while UNI50 clusters are further from UNI0 and show weaker alignment with these performance vectors. UNI75 is positioned far from UNI0. The heatmap illustrates the normalized expression (range 0–1) of all variables across dietary groups (Figure 4B). UNI0 shows the highest normalized values (red) for most indicators. In contrast, UNI75 displayed high‐intensity values for RBC and whole‐body composition associated with protein and ash. The UNI25 group exhibited moderately high values for the amino acid‐related digestibility parameters. UNI50 showed increased intensities for lipid‐related parameters, including EPA, DHA, and whole‐body lipid content.

**Figure 4:**
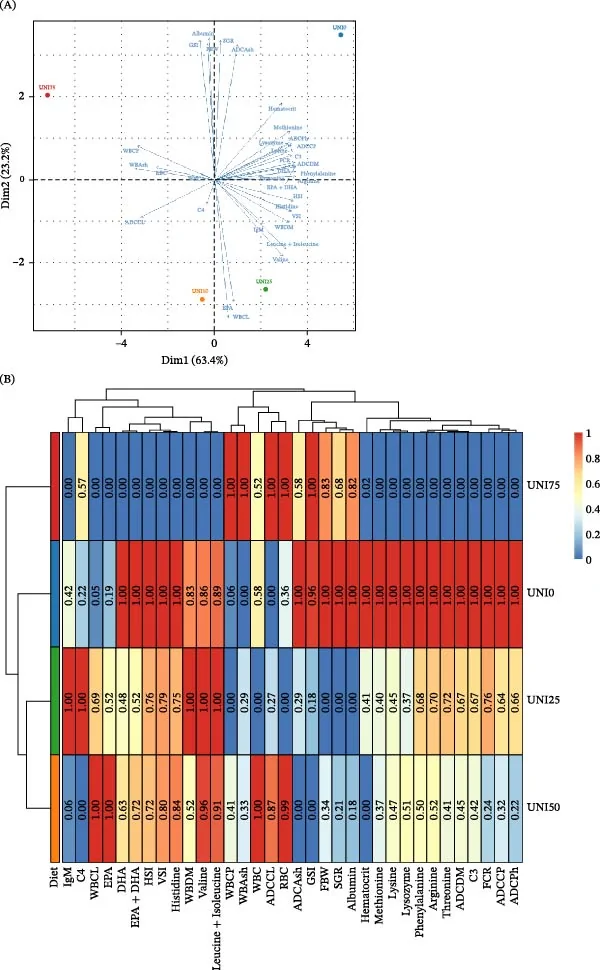
Overall effect of experimental diets on tested biological indices of Siberian sturgeon. (A) Principal component analysis (PCA) biplot: arrows represent variable loadings, and points represent individual samples colored by diet, (B) heatmap with hierarchical clustering of standardized biological indices across experimental diets: clustering of both diets (rows) and variables (columns) reveals patterns of similarity and differences. Heatmap values were normalized to a 0–1 scale for each variable, with 0 representing the minimum and 1 representing the maximum value observed for that variable.

## 4. Discussion

Growth performance and feed utilization parameters were not significantly affected by the inclusion of dietary MCP. These results are consistent with findings in juvenile rainbow trout (*Oncorhynchus mykiss*), in which partial replacement of fishmeal with MCP did not impair growth performance [20]. Similarly, Jintasataporn et al. [44] reported no adverse effects on growth, feed utilization, or survival in Pacific white shrimp fed diets in which fishmeal was completely replaced with MCP. On the other hand, the potential inclusion levels of MCPs that sustain growth in aquaculture species vary considerably across studies and are strongly influenced by factors such as the specific type of MCP, the substrates used to produce it, and the physiological characteristics of the target species [45]. In the present study, Siberian sturgeon showed good acceptance of unconventional protein sources in their diets, a finding that aligns with previous research [3, 8, 9, 12].

The elevated HSI observed in MCP‐fed fish may reflect altered hepatic nutrient partitioning or metabolic activity. However, HSI alone cannot distinguish between increased metabolic activity and lipid accumulation. The absence of significant differences in the evaluated histological parameters, including steatosis, hyperemia, and intercellular edema, argues against substantial hepatic structural damage under the conditions of the present experiment. Therefore, the increase in HSI should be interpreted alongside changes in hepatic lipid classes and metabolic gene expression, rather than as evidence of pathological liver enlargement.

Dry matter, crude protein, and essential amino acids (phenylalanine and arginine) digestibility were significantly affected by dietary MCP. Similar reductions in nutrient digestibility have been reported in Japanese yellowtail, black sea bream (*Acanthopagrus schlegelii*), spotted seabass (*Lateolabrax maculatus*), and turbot (*Scophthalmus maximus*) fed MCP‐containing diets [17, 46–48]. The reduced digestibility observed in the present study is likely multifactorial rather than attributable to a single component of MCP. One possible contributing factor is the cellular structure of methanotrophic bacteria. Bacterial cell wall components, including peptidoglycan and other structural materials, may reduce the accessibility of intracellular nutrients to digestive enzymes, thereby limiting nutrient hydrolysis. This constraint is likely more pronounced in Siberian sturgeon juveniles, which have a limited capacity to utilize complex carbohydrates [49]. However, other characteristics of MCP may also influence nutrient availability. Although the experimental diets were formulated to be isonitrogenous, replacing fishmeal with MCP progressively changed the dietary protein source and amino acid composition. Differences in protein structure, amino acid availability, and the utilization of absorbed nitrogen may therefore have contributed to the lower apparent digestibility of protein and amino acids observed at higher MCP inclusion levels. This may be particularly relevant to phenylalanine and arginine, which showed significant reductions as MCP inclusion increased. Other MCP‐specific characteristics, including cellular nucleic acid content and processing conditions, may also affect nutrient availability and utilization. However, these factors were not independently quantified or experimentally manipulated in the present study, and their contribution to the observed digestibility responses therefore remains uncertain.

Interestingly, lipid digestibility increased with increasing MCP levels, suggesting that the lower protein and dry‐matter digestibility of MCP‐containing diets was not accompanied by a reduction in digestive capacity. One possible explanation is that changes in dietary lipid composition and bile‐dependent lipid digestion favored lipid hydrolysis and absorption. The progressive replacement of fishmeal was accompanied by changes in the proportions of rapeseed and fish oils in the diets, and these formulation changes should therefore also be considered when interpreting the increased lipid digestibility [50]. Enhanced lipid uptake may explain the elevated HSI observed in MCP‐fed groups, potentially due to lipid accumulation or increased hepatic metabolic activity [51–53].

Despite the absence of significant changes in hematological and serum biochemical parameters, replacement of fishmeal with MCP affected selected innate immune‐related parameters. Complement C3 and lysozyme activity decreased with increasing MCP replacement, particularly at the highest replacement level. In contrast, expression of the pro‐inflammatory cytokines *il-1*β and *tnf-α* and the chemokine *il-8* increased in the head kidney of MCP‐fed fish. These findings indicate that MCP influenced immune regulation, but they do not demonstrate a uniform enhancement or suppression of innate immunity. The biological interpretation of the cytokine response requires caution. *il-1*β and *tnf-α* are central mediators of innate immune signaling, while *il-8* contributes to leukocyte recruitment. Increased transcription of these genes can therefore indicate the activation of innate immune signaling, but cytokine transcription alone is insufficient to establish tissue inflammation or pathological immune activation [54, 55]. Indeed, the present study did not measure cytokine protein concentrations, inflammatory signaling activity, oxidative stress, intestinal barrier integrity, or other direct markers of inflammation. We therefore interpret these responses as evidence of immune activation or modulation rather than definitive evidence of low‐grade pathological inflammation. One plausible mechanism is the recognition of bacterial‐associated molecular structures by the fish’s innate immune system. Methanotroph bacterial meal has been shown in other fish species to affect innate immune signaling, including cytokine and pattern‐recognition pathways. In largemouth bass, for example, *M. capsulatus* bacterial meal increased the expression of *tlr2*, *RelA*, *tnf-α*, and *il-1*β, with the response depending on the dietary inclusion level [22]. Conversely, in spotted seabass exposed to high‐soybean‐meal diets, *M. capsulatus* supplementation decreased *tnf-α*, *il-1*β, and *il-8* while increasing anti‐inflammatory genes such as *tgfβ*, *il-4*, and *il-10* [47]. These contrasting responses indicate that the immunological effects of MCP are strongly context‐dependent and may depend on the background diet, inclusion level, fish species, and physiological state.

The current data also do not support the conclusion that the observed cytokine response was specifically caused by lipopolysaccharides or peptidoglycan through Toll‐like receptor signaling [54, 55]. Although bacterial molecular patterns can activate innate immune pathways, the concentrations of these components in the MCP used here, and the activation of specific receptors, were not measured. The absence of significant changes in hematological and serum biochemical parameters, and of significant treatment‐related differences in the hepatic histological parameters evaluated, provides no evidence of overt systemic or hepatic pathology. Consequently, the increased cytokine expression should not be interpreted as unequivocal immunostimulation or as adverse inflammation.

Changes in hepatic cholesterol and cholesteryl ester, together with alterations in the expression of selected lipid‐metabolism‐related genes, suggest that increasing MCP inclusion influenced hepatic lipid metabolic processes [56, 57]. Hepatic cholesterol decreased with increasing MCP replacement, whereas cholesteryl ester concentrations increased at higher replacement levels. This combination is biologically relevant because hepatic cholesterol is regulated through coordinated processes involving endogenous cholesterol synthesis, uptake from circulating lipoproteins, intracellular esterification, membrane incorporation, conversion to bile acids, and biliary excretion [58, 59]. Evidence from other aquatic animals supports the broader concept that MCP can affect lipid metabolism. Dietary MCP altered lipid synthesis and lipolysis pathways in Pacific white shrimp [60], demonstrating that methanotroph protein can influence lipid metabolic regulation beyond its role as a protein source. This study supports the biological plausibility of altered sterol homeostasis in the present experiment, but does not establish the specific pathway responsible in Siberian sturgeon.

Histological examination showed no significant differences among dietary treatments in hyperemia, intercellular edema, or steatosis, indicating that MCP inclusion did not produce detectable treatment‐related alterations in the liver parameters evaluated under the present experimental conditions. This aligns with studies in other fish species, in which bacterial proteins modified liver metabolism without causing histopathology [61]. Nevertheless, intestinal morphology was not assessed in this study, which is a limitation because the intestine is the primary site of interaction with dietary MCP. Future studies should therefore combine hepatic assessment with intestinal histology and markers of intestinal barrier integrity to provide a more comprehensive evaluation of the physiological effects of MCP.

Gene expression analyses revealed significant metabolic modulation in response to MCP inclusion. *pck1* was downregulated in UNI25 and UNI75, whereas *hexok4* was upregulated in MCP‐fed groups, indicating changes in carbohydrate and energy metabolism. The hepatic accumulation of cholesteryl esters further supports this lipogenic state [59, 62].

The pronounced upregulation of pro‐inflammatory cytokines *il-1*β, *tnf-α*, and *il-8* in all MCP‐fed groups indicates immune activation and possible low‐grade inflammation induced by bacterial protein inclusion. *il-1*β and *tnf*‐α are central mediators of innate immune responses, promoting leukocyte recruitment and antimicrobial defenses. *il-8* specifically facilitates neutrophil chemotaxis to sites of inflammation [63]. These elevations likely result from the recognition of microbial‐associated molecular patterns, such as lipopolysaccharides or peptidoglycans present in MCP, triggering signaling through pattern recognition receptors in immune cells [64, 65]. However, these transcriptional changes should not be interpreted as direct evidence of inflammation, particularly in the absence of corresponding protein‐level measurements or other direct markers of inflammatory activity. The mechanisms underlying these immune‐related responses remain to be elucidated.

The expression of *ghr* was higher in UNI25, while *gh* and *rpl6* expression declined in MCP‐fed groups, suggesting alterations in the somatotropic axis at higher MCP inclusion levels [66–68]. Growth hormone and its receptor regulate growth, protein metabolism, lipid metabolism, and nutrient partitioning in fish [69]; therefore, changes in these transcripts could represent an adaptive response to the altered nutrient availability. Nevertheless, because circulating growth hormone, *ifg-i*, or downstream signaling activity was not measured, the functional significance of these transcriptional changes remains uncertain. Together, these findings indicate that MCP inclusion affected selected metabolic and endocrine pathways in Siberian sturgeon.

Ash content and fatty acid composition were significantly affected by the inclusion of MCP. Ash content increased with increasing MCP inclusion, whereas saturated and monounsaturated fatty acids also showed significant dietary responses. These changes may partly reflect differences in the mineral and fatty‐acid composition of MCP‐containing diets rather than direct metabolic effects of MCP. Dietary replacement changes not only the protein source but also the relative contribution of associated minerals and lipid sources, including rapeseed oil and fish oil. The observed changes in SFA and MUFA suggest that replacing fishmeal with MCP can influence the partitioning or deposition of individual fatty acid classes even when total dietary lipid is maintained. However, the biological significance of these changes should be interpreted with caution, as the mechanisms underlying the altered proportions of SFA and MUFA were not directly investigated. Changes in tissue fatty acid composition can reflect differences in dietary fatty acid composition as well as selective incorporation, oxidation, elongation, desaturation, and endogenous synthesis [57]. The absence of significant differences in EPA and DHA is also consistent with the dietary strategy used in this experiment, in which the lipid sources were adjusted to maintain their supply across the experimental diets. The fatty acid composition of farmed fish generally reflects dietary fatty acid supply, although the degree of dietary fatty acid incorporation varies according to fatty acid type, species, tissue, and other experimental conditions [70]. In Siberian sturgeon, Nieminen et al. [71] reported that tissue fatty acid profiles differed among tissues and from the dietary profile, with accumulation of particular highly unsaturated fatty acids, demonstrating that tissue deposition reflects both dietary supply and endogenous metabolic processes. Therefore, the present results suggest that the MCP‐associated changes in SFA and MUFA did not extend to the major long‐chain n‐3 fatty acids EPA and DHA when their dietary supply was maintained.

In this study, the intestinal microbiota of sturgeon were dominated by three major bacterial phyla—Fusobacteriota, Firmicutes, and Proteobacteria—irrespective of dietary treatment. This microbial profile is consistent with previous characterizations of sturgeon intestinal microbiota, where these phyla are frequently reported as core constituents of the gut community [72, 73]. Importantly, however, MCP did not significantly alter phylum‐level or genus‐level relative abundance among dietary treatments. Similarly, alpha‐diversity and overall beta‐diversity did not differ significantly among diets. These findings indicate that fishmeal replacement with MCP did not cause a major restructuring of the established intestinal microbial community during the 70‐day experiment.

The temporal shifts in Bacteroidota and Crenarchaeota may nevertheless reflect changes in substrate availability associated with feeding and environmental adaptation. These taxa have been associated with proteolytic and lipid metabolic capacities and may have responded to the altered nutrient profile of MCP diets [73]. Because the present study used 16S rRNA profiling rather than metagenomics, metatranscriptomics, or metabolomics, we cannot determine whether these taxonomic changes altered microbial metabolic pathways or host–microbe interactions. Therefore, the most appropriate interpretation is that MCP replacement preserved the overall microbial community structure, while the intestinal microbiota underwent temporal changes in specific taxa during the feeding period.

*Cetobacterium* and *Clostridium* were the dominant genera across all experimental groups. *Cetobacterium*, a common genus in freshwater fish, produces vitamin B12 and short‐chain fatty acids, particularly acetate, which can contribute to host energy metabolism and gut health [74]. *Clostridium* includes both commensal and opportunistic species and is associated with protein fermentation [75]; its persistence across diets may be linked to the high protein content of all feeds, including those with MCP. This study observed a significant change in the overall genus‐level composition between pre‐ and post‐feeding samples, suggesting a time‐dependent microbial shift rather than a diet‐specific response. This finding is consistent with previous studies in lake sturgeon, which reported temporal variability in gut microbiota composition [76]. The maturation of the gut microbiota or adaptation to the rearing environment and feeding regime may underlie this community restructuring. No overt dysbiosis or pathogen overgrowth was detected, indicating that the observed compositional shifts may reflect functional adaptation.

## 5. Conclusion

The results suggest that a 25% replacement of fishmeal with MCP (5.25% MCP inclusion) yielded a physiological profile broadly comparable to that of the fishmeal control. In contrast, higher replacement levels were associated with increasingly evident changes in nutrient utilization and selected immune‐related and metabolic responses. These findings indicate that MCP has potential as a partial replacement for fishmeal in Siberian sturgeon, but the biological consequences depend on the replacement level. The ability to produce protein‐rich microbial biomass from methane, together with the development of commercially available MCP products, indicates that this technology has potential for industrial‐scale application. Previous studies have also demonstrated the feasibility of incorporating MCP into diets for several aquaculture species. However, commercial application should be considered in relation to both nutritional performance and production economics. In the present study, MCP inclusion did not impair growth performance, but higher inclusion levels were associated with reduced digestibility of several nutrients and alterations in immune‐related and hepatic metabolic responses. Further optimization of MCP processing and diet formulation may be necessary to improve nutrient utilization at higher inclusion levels. Although MCP production has potential for large‐scale manufacture, its economic competitiveness will ultimately depend on production efficiency, methane availability, processing costs, product consistency, and the market price relative to conventional protein sources. These economic and scalability aspects were not directly evaluated in the present study and therefore require dedicated techno‐economic and life‐cycle assessments before definitive conclusions regarding commercial competitiveness can be made.

## Author Contributions

**Hien Van Doan:** conceptualization, investigation, formal analysis, writing – original draft. **Pavel Hyrsl:** investigation, methodology. **Eliska Zuskova:** investigation, methodology. **Roey Angel:** investigation, methodology. **Ales Tomcala:** investigation, methodology. **Tram Thi Nguyen:** data curation, methodology. **Mahyar Zare:** investigation, methodology. **Ghasem Rashidian:** investigation, methodology. **Vlastimil Stejskal:** supervision, writing – review and editing. **Hung Quang Tran:** conceptualization, funding acquisition, resources, supervision, writing – original draft, writing – review and editing.

## Funding

The present study is financially supported by the Ministry of Agriculture of the Czech Republic (NAZV project, Grant QL24010346). Part of the work was carried out with the support of VVI CENAKVA Research Infrastructure (Grant ID 90238, MEYS CR, 2023–2026). This work was also partially supported by the Chiang Mai University, Thailand.

## Conflicts of Interest

The authors declare no conflicts of interest.

## Supporting Information

Additional supporting information can be found online in the Supporting Information section.

## Supporting information


**Supporting Information 1** Table S1: Lipid classes (% total lipids) in experimental diets. Table S2: Fatty acid profile (% of total fatty acids) of experimental diets. Table S3: The histopathological indices of liver of Siberian sturgeon fed experimental diets for 70 days. Table S4: The sequences of the primers used in the RT‐PCR of liver samples. Table S5: The sequences of the primers used in the RT‐PCR of head kidney samples. Table S6: The sequences of the primers used in the RT‐PCR of intestine samples. Table S7: Wilcoxon signed‐rank test results for phyla level. Table S8: Wilcoxon signed‐rank test results for genus level.

**Supporting Information 2** Figure S1: Liver of Siberian sturgeon (*Acipenser baerii*) fed with dietary MCP, H&E, 400x. (A) UNI0; (B) UNI25; (C) UNI50; (D) UNI75. The arrowheads indicate vessels; the arrows mark exemplary lipidic hepatocytes; the asterisk mark extensive hyperemic place. Figure S2: Alpha diversity measured by Shannon and Simpson indexes. Data are shown as box plots (*n* = 9). The horizontal line indicates the median, and the whiskers indicate the lowest and highest points within the lower or upper quartile interquartile ranges.

## Data Availability

Raw sequencing data and metadata for all samples included in this study have been uploaded to the sequence read archive (SRA) database of NCBI (https://www.ncbi.nlm.nih.gov/sra) and can be accessed with the Bioproject accession number, PRJNA1276463.
