# Supplementary material for: Effects of Partial Fishmeal Replacement With Methanotrophic Bacterial Protein (Methylococcus capsulatus) in Siberian Sturgeon (Acipenser baerii) on Growth Performance, Nutrient Utilization, Body Composition, Immune Responses, Liver Histology, Gene Expression, and Gut Microbiota
Source: Aquac Nutr. 2026 Nov 2;2026:3788480. doi: 10.1155/anu/3788480 (PMC13728454; doi:10.1155/anu/3788480)
Supplement: Supplementary file 1 [file ANU-2026-3788480-s001.docx]

# Supplemental Table 1 Lipid classes (% total lipids) in experimental diets.

| Lipid classes | Experimental diets | | | | SEM |
| --- | --- | --- | --- | --- | --- |
|  | UNI0 | UNI25 | UNI50 | UNI75 |  |
| Sphingomyelin | 1.22 | 0.77 | 0.95 | 0.99 | 0.10 |
| Phosphatidylcholine | 10.80 | 8.10 | 8.63 | 8.29 | 0.49 |
| Phosphatidylinositol | 4.52 | 3.78 | 4.37 | 4.54 | 0.20 |
| Cardiolipin | 5.80 | 4.76 | 5.34 | 5.83 | 0.25 |
| Phosphatidylethanolamine | 4.21 | 21.69 | 10.57 | 12.42 | 3.48 |
| **Total polar lipids** | **26.55** | **39.11** | **29.86** | **32.07** | **4.52** |
| Free fatty acid | 5.15 | 4.21 | 4.94 | 4.75 | 0.17 |
| Cholesterol | 14.76 | 12.24 | 13.82 | 14.02 | 0.47 |
| Triacylglycerol | 26.14 | 24.35 | 27.03 | 26.59 | 1.17 |
| Cholesteryl | 11.48 | 6.59 | 9.66 | 6.31 | 1.17 |
| **Total neutral lipids** | **57.52** | **47.40** | **55.45** | **51.68** | **2.98** |
| **Others** | **15.94** | **13.50** | **14.69** | **16.26** | **0.67** |

SEM: Standard error of means

# Supplemental Table 2 Fatty acid profile (% of total fatty acids) of experimental diets.

| **Fatty acids** | **Experimental diets** | | | | **SEM** |
| --- | --- | --- | --- | --- | --- |
|  | **UNI0** | **UNI25** | **UNI50** | **UNI75** |  |
| C14:0 | 1.79 | 1.86 | 1.88 | 1.89 | 0.013 |
| C14:1 | 0.05 | 0.08 | 0.09 | 0.11 | 0.007 |
| C16:0 | 11.16 | 12.71 | 14.11 | 16.16 | 0.556 |
| C16:1 | 1.66 | 2.58 | 3.62 | 4.77 | 0.351 |
| C18:0 | 2.14 | 2.23 | 2.33 | 2.35 | 0.026 |
| C18:1n9 (OA) | 38.68 | 36.20 | 34.17 | 30.62 | 0.894 |
| C18:1n7 | 2.59 | 2.65 | 2.74 | 2.78 | 0.023 |
| C18:2n6 (LA) | 25.06 | 24.99 | 24.51 | 25.36 | 0.120 |
| C18:3n3 | 5.19 | 5.08 | 5.05 | 4.92 | 0.033 |
| C20:0 | 0.36 | 0.33 | 0.31 | 0.27 | 0.010 |
| C20:1n9 | 3.55 | 3.47 | 3.33 | 3.15 | 0.047 |
| C20:2n6 | 0.30 | 0.37 | 0.45 | 0.52 | 0.025 |
| C20:4n6 | 0.14 | 0.15 | 0.16 | 0.15 | 0.002 |
| C20:3n3 | 0.12 | 0.14 | 0.19 | 0.21 | 0.012 |
| C22:0 | 0.20 | 0.19 | 0.18 | 0.15 | 0.007 |
| C22:1n9 | 0.34 | 0.35 | 0.36 | 0.36 | 0.003 |
| C20:5n3 (EPA) | 2.07 | 2.06 | 2.04 | 1.94 | 0.016 |
| C24:0 | 0.12 | 0.11 | 0.10 | 0.06 | 0.009 |
| C24:1n9 | 0.31 | 0.30 | 0.29 | 0.27 | 0.005 |
| C22:5n6 | 0.40 | 0.48 | 0.56 | 0.61 | 0.024 |
| C22:6n3 (DHA) | 3.74 | 3.66 | 3.54 | 3.34 | 0.047 |
| SFA | 15.78 | 17.43 | 18.90 | 20.88 | 0.569 |
| MUFA | 47.18 | 45.63 | 44.60 | 42.06 | 0.569 |
| PUFA | 37.04 | 36.94 | 36.51 | 37.06 | 0.101 |
| n-3 | 11.13 | 10.95 | 10.83 | 10.42 | 0.081 |
| n-6 | 25.91 | 25.99 | 25.68 | 26.65 | 0.130 |
| n-3/n-6 | 0.43 | 0.42 | 0.42 | 0.39 | 0.005 |
| EPA+DHA | 5.82 | 5.72 | 5.59 | 5.28 | 0.063 |

OA: Oleic acid; LA: linoleic acid; EPA: eicosapentaenoic acid; DHA: docosahexaenoic acid; SFA: saturated fatty acids; MUFA: monounsaturated fatty acids; PUFA: polyunsaturated fatty acids

# Supplemental Table 3 The histopathological indices of liver of Siberian sturgeon fed experimental diets for 70 days.

| **Index** | **Experimental diets** | | | | ***Kruskal-Wallis***  ***P_value*** |
| --- | --- | --- | --- | --- | --- |
|  | **UNI0** | **UNI25** | **UNI50** | **UNI75** |  |
| Hyperemia | 2.11 ± 0.20 | 2.00 ± 0.24 | 2.00 ± 0.29 | 2.44 ± 0.18 | 0.495 |
| Intercellular oedema | 1.67 ± 0.29 | 1.78 ± 0.22 | 1.89 ± 0.20 | 1.89 ± 0.31 | 0.878 |
| Steatosis | 1.89 ± 0.26 | 2.00 ± 0.24 | 2.33 ± 0.24 | 2.33 ± 0.24 | 0.443 |

Data are presented as the mean (n = 3 fish/tank or 9 fish/group), values in the same row with different superscripts are significantly different (P < 0.05)

# Supplemental Table 4. The sequences of the primers used in the RT-PCR of liver samples.

| **Gene** | **Primer sequences**  **(5’→3’)** | **Product size**  **(bp)** | **Temperature**  **(℃)** | **Reference** |
| --- | --- | --- | --- | --- |
| *pck1* | F-CCTGCACATCTGCGATGGAT  R-TACATGGTGCGCCCTTTCAT | 133 | 58.9 | Yang et al. (2024)* |
| *fatp6* | F-ACCTCAGGAACCACAGGTCT  R-GCAGCACTGTGATATAGAGGCA | 140 | 60.0 |  |
| *gck* | F-ACAAGCTACACCCCAATTTCCA  R-TTGCAAGCCACTGCTGAGAT | 136 | 61.2 |  |
| *hexok 4* | F-GAGACCTCCACCAACAGTGG  R-CGGTCCCCAGAATCACTTTCA | 200 | 60.4 |  |
| *fas* | F-CTCAGTACTGTGCAAGCTGGA  R-CCCATTGCTAGACAAAACCTCAA | 205 | 60.8 |  |
| *acc* | F-CGGAGGCTGCTCCTGGAATA  R-TCCCAGAGGTACGCCTTGAC | 137 | 61.0 |  |
| *igf1* | F-CTCCTGTGTGTTCTGTGCCT  R-CCATAGCCTGTTGGTTTGTTGAA | 134 | 58.9 |  |
| *caspase 9* | F-CATGGCACAGAGGTGAACCA  R-CTTTCCCAAGCTCCTCCCTG | 274 | 59.5 |  |
| *gadd45β* | F-GCGAAGCTGATGAATGTCGAT  R-GGACACTCGCAGGATGTTGA | 150 | 59.7 |  |
| *β-actin* | F-GCCAGGGCTGTGATTTCTTT  R-GCTGCTTCCTCTTCCTCCCT | 275 | 57.6 |  |

F=forward; R=reverse; *pck1*=phosphoenolpyruvate carboxykinase 1; *fatp6*=fatty acid transport protein; *gck*=glucokinase (hexokinase 4); *hexok 4*=hexokinase (Hexok); *fas*=fatty acid synthase; *acc*=acetyl-CoA carboxylase; *igf1*=insulin-like growth factor 1; *caspase-9*=cysteinyl aspartate specific proteinase 9; *gadd45β*=growth arrest and DNA damage gene 45β

*Yang, S., Yan, C., Pang, X., Shao, W., Xu, Z., Li, D., Xu, W., Zhang, Z., Su, B., Li, Y., Wu, J., Huang, X., Luo, W., Du, X., 2024. Transcriptome analysis reveals high concentration of resveratrol promotes lipid synthesis and induces apoptosis in Siberian sturgeon (*Acipenser baerii*). BMC Genomics 25(1), 821. https://doi.org/10.1186/s12864-024-10698-0

# Supplemental Table 5 The sequences of the primers used in the RT-PCR of head kidney samples.

| **Gene** | **Primer sequences**  **(5’→3’)** | **Product size**  **(bp)** | **Temperature**  **(℃)** | **Reference** |
| --- | --- | --- | --- | --- |
| *il-1β* | F- TGATGAACGAGCTGGATGGG  R- GCTGGGTCTGCGGTATGTAG | 57.1 | 114 | Chen et al. (2023) |
| *tnf-α* | F-TCGCCGGACTTCACAATAGG  R-GCTTGCTCGCCAGTTGTTTT | 58.9 | 114 |  |
| *il-8* | F- GGTGCAAATTCTCCCAGCAAA  R- AACTCCACTCCCAAAGGAGC | 61.4 | 107 |  |
| *tgf-β* | F- ATTCAGAACTATAAGACCCCCC  R- CGGAAGTCAATGTAAAGAGGC | 63.3 | 161 |  |
| *β-actin* | F- GTTGGTATGGGACAGAAGGACA  R- CCAGTTGGTAACAATGCCGT | 61.4 | 105 |  |

*il-1β*=interleukin-1β; *tnf-α*=tumor necrosis factor α; *tgf-β*=transforming growth factor β

* Chen, D., Chen, Y., Lu, L., Zhu, H., Zhang, X., Huang, X., Li, Z., Ouyang, P., Zhang, X., Li, L., Geng, Y., 2023. Transcriptome revealed the macrophages inflammatory response mechanism and nod-like receptor characterization in Siberian sturgeon (*Acipenser baerii*). Int J Mol Sci 24(11). https://doi.org/10.3390/ijms24119518.

# Supplemental Table 6 The sequences of the primers used in the RT-PCR of intestine samples.

| **Gene** | **Primer sequences**  **(5’→3’)** | **Product size**  **(bp)** | **Temperature**  **(℃)** | **Reference** |
| --- | --- | --- | --- | --- |
| *ghr* | F-CCAAGGTGACACGTCGAGAT  R-GTTTTTGCAACAGGTGCCCA | 159 | 59 | Ramezani et al. (2021) |
| *igf* | F-CCTGTGTGTTCTGTGCCTGA  R-CTGGCTCCATAGCCTGTTGG | 134 | 60 |  |
| *gh* | F-GAGTCTGGGGCCTACCCTAT  R-TGACGCTGCTCATCTGGAAC | 137 | 59 |  |
| *rpl6* | F-TGCCTCGGTATTACCCTACC  R- AGGACAGTGCCAGGAGTGAT | 115 | 59 |  |

*ghr*=growth hormone receptor; *igf*=insulin-like growth factor; *gh*=growth hormone; *rpl6*=ribosomal protein L6

*Ramezani, F., Shekarabi, S.P.H., Mehrgan, M.S., Foroudi, F., Islami, H.R., 2021. Supplementation of Siberian sturgeon (*Acipenser baerii*) diet with barberry (*Berberis vulgaris*) fruit extract: Growth performance, hemato-biochemical parameters, digestive enzyme activity, and growth-related gene expression. Aquaculture 540, 736750. https://doi.org/https://doi.org/10.1016/j.aquaculture.2021.736750.

# Supplemental Table 7 Wilcoxon signed-rank test results for phyla level.

| **Phylum** | **Group_Comparison** | ***p_value*** | ***p_adj*** |
| --- | --- | --- | --- |
| Actinobacteriota | INI vs UNI0 | 0.263 | 0.526 |
| Actinobacteriota | INI vs UNI25 | 0.860 | 0.860 |
| Actinobacteriota | INI vs UNI50 | 0.238 | 0.526 |
| Actinobacteriota | INI vs UNI75 | 0.479 | 0.639 |
| Bacteroidota | INI vs UNI0 | 0.002 | 0.004 |
| Bacteroidota | INI vs UNI25 | 0.020 | 0.020 |
| Bacteroidota | INI vs UNI50 | 0.010 | 0.013 |
| Bacteroidota | INI vs UNI75 | 0.002 | 0.004 |
| Chloroflexi | INI vs UNI0 | 0.765 | 0.765 |
| Chloroflexi | INI vs UNI25 | 0.201 | 0.637 |
| Chloroflexi | INI vs UNI50 | 0.483 | 0.644 |
| Chloroflexi | INI vs UNI75 | 0.319 | 0.637 |
| Crenarchaeota | INI vs UNI0 | 0.029 | 0.029 |
| Crenarchaeota | INI vs UNI25 | 0.029 | 0.029 |
| Crenarchaeota | INI vs UNI50 | 0.029 | 0.029 |
| Crenarchaeota | INI vs UNI75 | 0.029 | 0.029 |
| Cyanobacteria | INI vs UNI0 | 0.860 | 0.860 |
| Cyanobacteria | INI vs UNI25 | 0.860 | 0.860 |
| Cyanobacteria | INI vs UNI50 | 0.768 | 0.860 |
| Cyanobacteria | INI vs UNI75 | 0.637 | 0.860 |
| Firmicutes | INI vs UNI0 | 0.517 | 0.517 |
| Firmicutes | INI vs UNI25 | 0.444 | 0.517 |
| Firmicutes | INI vs UNI50 | 0.444 | 0.517 |
| Firmicutes | INI vs UNI75 | 0.517 | 0.517 |
| Fusobacteriota | INI vs UNI0 | 0.596 | 0.795 |
| Fusobacteriota | INI vs UNI25 | 0.316 | 0.754 |
| Fusobacteriota | INI vs UNI50 | 1.000 | 1.000 |
| Fusobacteriota | INI vs UNI75 | 0.377 | 0.754 |
| Myxococcota | INI vs UNI0 | 0.765 | 0.765 |
| Myxococcota | INI vs UNI25 | 0.276 | 0.368 |
| Myxococcota | INI vs UNI50 | 0.276 | 0.368 |
| Myxococcota | INI vs UNI75 | 0.276 | 0.368 |
| Planctomycetota | INI vs UNI0 | 0.866 | 1.000 |
| Planctomycetota | INI vs UNI25 | 0.921 | 1.000 |
| Planctomycetota | INI vs UNI50 | 1.000 | 1.000 |
| Planctomycetota | INI vs UNI75 | 0.765 | 1.000 |
| Proteobacteria | INI vs UNI0 | 0.680 | 0.860 |
| Proteobacteria | INI vs UNI25 | 0.860 | 0.860 |
| Proteobacteria | INI vs UNI50 | 0.175 | 0.526 |
| Proteobacteria | INI vs UNI75 | 0.263 | 0.526 |
| Unclassified | INI vs UNI0 | 0.276 | 0.276 |
| Unclassified | INI vs UNI25 | 0.276 | 0.276 |
| Unclassified | INI vs UNI50 | 0.276 | 0.276 |
| Unclassified | INI vs UNI75 | 0.276 | 0.276 |
| Verrucomicrobiota | INI vs UNI0 | 0.765 | 0.921 |
| Verrucomicrobiota | INI vs UNI25 | 0.276 | 0.921 |
| Verrucomicrobiota | INI vs UNI50 | 0.921 | 0.921 |
| Verrucomicrobiota | INI vs UNI75 | 0.765 | 0.921 |

# Supplemental Table 8 Wilcoxon signed-rank test results for genus level.

| **Genus** | **Group_Comparison** | ***p_value*** | ***p_adj*** |
| --- | --- | --- | --- |
| *Acidaminococcus* | INI vs UNI0 | 9.85 × 10^-21^ | 3.94 × 10^-20^ |
| *Acidaminococcus* | INI vs UNI25 | 3.34 × 10^-14^ | 3.34 × 10^-14^ |
| *Acidaminococcus* | INI vs UNI50 | 2.95 × 10^-19^ | 5.9 × 10^-19^ |
| *Acidaminococcus* | INI vs UNI75 | 1.56 × 10^-18^ | 2.08 × 10^-18^ |
| *Acinetobacter* | INI vs UNI0 | 9.85 × 10^-21^ | 3.94 × 10^-20^ |
| *Acinetobacter* | INI vs UNI25 | 3.34 × 10^-14^ | 3.34 × 10^-14^ |
| *Acinetobacter* | INI vs UNI50 | 2.95 × 10^-19^ | 5.9 × 10^-19^ |
| *Acinetobacter* | INI vs UNI75 | 1.56 × 10^-18^ | 2.08 × 10^-18^ |
| *Actinomyces* | INI vs UNI0 | 9.85 × 10^-21^ | 3.94 × 10^-20^ |
| *Actinomyces* | INI vs UNI25 | 3.34 × 10^-14^ | 3.34 × 10^-14^ |
| *Actinomyces* | INI vs UNI50 | 2.95 × 10^-19^ | 5.9 × 10^-19^ |
| *Actinomyces* | INI vs UNI75 | 1.56 × 10^-18^ | 2.08 × 10^-18^ |
| *Actinoplanes* | INI vs UNI0 | 9.85 × 10^-21^ | 3.94 × 10^-20^ |
| *Actinoplanes* | INI vs UNI25 | 3.34 × 10^-14^ | 3.34 × 10^-14^ |
| *Actinoplanes* | INI vs UNI50 | 2.95 × 10^-19^ | 5.9 × 10^-19^ |
| *Actinoplanes* | INI vs UNI75 | 1.56 × 10^-18^ | 2.08 × 10^-18^ |
| *Actinotalea* | INI vs UNI0 | 9.85 × 10^-21^ | 3.94 × 10^-20^ |
| *Actinotalea* | INI vs UNI25 | 3.34 × 10^-14^ | 3.34 × 10^-14^ |
| *Actinotalea* | INI vs UNI50 | 2.95 × 10^-19^ | 5.9 × 10^-19^ |
| *Actinotalea* | INI vs UNI75 | 1.56 × 10^-18^ | 2.08 × 10^-18^ |
| *Aeriscardovia* | INI vs UNI0 | 9.85 × 10^-21^ | 3.94 × 10^-20^ |
| *Aeriscardovia* | INI vs UNI25 | 3.34 × 10^-14^ | 3.34 × 10^-14^ |
| *Aeriscardovia* | INI vs UNI50 | 2.95 × 10^-19^ | 5.9 × 10^-19^ |
| *Aeriscardovia* | INI vs UNI75 | 1.56 × 10^-18^ | 2.08 × 10^-18^ |
| *Aeromonas* | INI vs UNI0 | 9.85 × 10^-21^ | 3.94 × 10^-20^ |
| *Aeromonas* | INI vs UNI25 | 3.34 × 10^-14^ | 3.34 × 10^-14^ |
| *Aeromonas* | INI vs UNI50 | 2.95 × 10^-19^ | 5.9 × 10^-19^ |
| *Aeromonas* | INI vs UNI75 | 1.56 × 10^-18^ | 2.08 × 10^-18^ |
| *Agrilactobacillus* | INI vs UNI0 | 9.85 × 10^-21^ | 3.94 × 10^-20^ |
| *Agrilactobacillus* | INI vs UNI25 | 3.34 × 10^-14^ | 3.34 × 10^-14^ |
| *Agrilactobacillus* | INI vs UNI50 | 2.95 × 10^-19^ | 5.9 × 10^-19^ |
| *Agrilactobacillus* | INI vs UNI75 | 1.56 × 10^-18^ | 2.08 × 10^-18^ |
| *Amnipila* | INI vs UNI0 | 9.85 × 10^-21^ | 3.94 × 10^-20^ |
| *Amnipila* | INI vs UNI25 | 3.34 × 10^-14^ | 3.34 × 10^-14^ |
| *Amnipila* | INI vs UNI50 | 2.95 × 10^-19^ | 5.9 × 10^-19^ |
| *Amnipila* | INI vs UNI75 | 1.56 × 10^-18^ | 2.08 × 10^-18^ |
| *Anaeromyxobacter* | INI vs UNI0 | 9.85 × 10^-21^ | 3.94 × 10^-20^ |
| *Anaeromyxobacter* | INI vs UNI25 | 3.34 × 10^-14^ | 3.34 × 10^-14^ |
| *Anaeromyxobacter* | INI vs UNI50 | 2.95 × 10^-19^ | 5.9 × 10^-19^ |
| *Anaeromyxobacter* | INI vs UNI75 | 1.56 × 10^-18^ | 2.08 × 10^-18^ |
| *Anaerosalibacter* | INI vs UNI0 | 9.85 × 10^-21^ | 3.94 × 10^-20^ |
| *Anaerosalibacter* | INI vs UNI25 | 3.34 × 10^-14^ | 3.34 × 10^-14^ |
| *Anaerosalibacter* | INI vs UNI50 | 2.95 × 10^-19^ | 5.9 × 10^-19^ |
| *Anaerosalibacter* | INI vs UNI75 | 1.56 × 10^-18^ | 2.08 × 10^-18^ |
| *Aquicella* | INI vs UNI0 | 9.85 × 10^-21^ | 3.94 × 10^-20^ |
| *Aquicella* | INI vs UNI25 | 3.34 × 10^-14^ | 3.34 × 10^-14^ |
| *Aquicella* | INI vs UNI50 | 2.95 × 10^-19^ | 5.9 × 10^-19^ |
| *Aquicella* | INI vs UNI75 | 1.56 × 10^-18^ | 2.08 × 10^-18^ |
| *Arcanobacterium* | INI vs UNI0 | 9.85 × 10^-21^ | 3.94 × 10^-20^ |
| *Arcanobacterium* | INI vs UNI25 | 3.34 × 10^-14^ | 3.34 × 10^-14^ |
| *Arcanobacterium* | INI vs UNI50 | 2.95 × 10^-19^ | 5.9 × 10^-19^ |
| *Arcanobacterium* | INI vs UNI75 | 1.56 × 10^-18^ | 2.08 × 10^-18^ |
| *Atopobium* | INI vs UNI0 | 9.85 × 10^-21^ | 3.94 × 10^-20^ |
| *Atopobium* | INI vs UNI25 | 3.34 × 10^-14^ | 3.34 × 10^-14^ |
| *Atopobium* | INI vs UNI50 | 2.95 × 10^-19^ | 5.9 × 10^-19^ |
| *Atopobium* | INI vs UNI75 | 1.56 × 10^-18^ | 2.08 × 10^-18^ |
| *Aurantimicrobium* | INI vs UNI0 | 9.85 × 10^-21^ | 3.94 × 10^-20^ |
| *Aurantimicrobium* | INI vs UNI25 | 3.34 × 10^-14^ | 3.34 × 10^-14^ |
| *Aurantimicrobium* | INI vs UNI50 | 2.95 × 10^-19^ | 5.9 × 10^-19^ |
| *Aurantimicrobium* | INI vs UNI75 | 1.56 × 10^-18^ | 2.08 × 10^-18^ |
| *Bacillus* | INI vs UNI0 | 9.85 × 10^-21^ | 3.94 × 10^-20^ |
| *Bacillus* | INI vs UNI25 | 3.34 × 10^-14^ | 3.34 × 10^-14^ |
| *Bacillus* | INI vs UNI50 | 2.95 × 10^-19^ | 5.9 × 10^-19^ |
| *Bacillus* | INI vs UNI75 | 1.56 × 10^-18^ | 2.08 × 10^-18^ |
| *Bacteroides* | INI vs UNI0 | 9.85 × 10^-21^ | 3.94 × 10^-20^ |
| *Bacteroides* | INI vs UNI25 | 3.34 × 10^-14^ | 3.34 × 10^-14^ |
| *Bacteroides* | INI vs UNI50 | 2.95 × 10^-19^ | 5.9 × 10^-19^ |
| *Bacteroides* | INI vs UNI75 | 1.56 × 10^-18^ | 2.08 × 10^-18^ |
| *Bauldia* | INI vs UNI0 | 9.85 × 10^-21^ | 3.94 × 10^-20^ |
| *Bauldia* | INI vs UNI25 | 3.34 × 10^-14^ | 3.34 × 10^-14^ |
| *Bauldia* | INI vs UNI50 | 2.95 × 10^-19^ | 5.9 × 10^-19^ |
| *Bauldia* | INI vs UNI75 | 1.56 × 10^-18^ | 2.08 × 10^-18^ |
| *Bifidobacterium* | INI vs UNI0 | 9.85 × 10^-21^ | 3.94 × 10^-20^ |
| *Bifidobacterium* | INI vs UNI25 | 3.34 × 10^-14^ | 3.34 × 10^-14^ |
| *Bifidobacterium* | INI vs UNI50 | 2.95 × 10^-19^ | 5.9 × 10^-19^ |
| *Bifidobacterium* | INI vs UNI75 | 1.56 × 10^-18^ | 2.08 × 10^-18^ |
| *Bosea* | INI vs UNI0 | 9.85 × 10^-21^ | 3.94 × 10^-20^ |
| *Bosea* | INI vs UNI25 | 3.34 × 10^-14^ | 3.34 × 10^-14^ |
| *Bosea* | INI vs UNI50 | 2.95 × 10^-19^ | 5.9 × 10^-19^ |
| *Bosea* | INI vs UNI75 | 1.56 × 10^-18^ | 2.08 × 10^-18^ |
| *Brevibacterium* | INI vs UNI0 | 9.85 × 10^-21^ | 3.94 × 10^-20^ |
| *Brevibacterium* | INI vs UNI25 | 3.34 × 10^-14^ | 3.34 × 10^-14^ |
| *Brevibacterium* | INI vs UNI50 | 2.95 × 10^-19^ | 5.9 × 10^-19^ |
| *Brevibacterium* | INI vs UNI75 | 1.56 × 10^-18^ | 2.08 × 10^-18^ |
| *Butyricicoccus* | INI vs UNI0 | 9.85 × 10^-21^ | 3.94 × 10^-20^ |
| *Butyricicoccus* | INI vs UNI25 | 3.34 × 10^-14^ | 3.34 × 10^-14^ |
| *Butyricicoccus* | INI vs UNI50 | 2.95 × 10^-19^ | 5.9 × 10^-19^ |
| *Butyricicoccus* | INI vs UNI75 | 1.56 × 10^-18^ | 2.08 × 10^-18^ |
| *Candidatus* | INI vs UNI0 | 9.85 × 10^-21^ | 3.94 × 10^-20^ |
| *Candidatus* | INI vs UNI25 | 3.34 × 10^-14^ | 3.34 × 10^-14^ |
| *Candidatus* | INI vs UNI50 | 2.95 × 10^-19^ | 5.9 × 10^-19^ |
| *Candidatus* | INI vs UNI75 | 1.56 × 10^-18^ | 2.08 × 10^-18^ |
| *Catenibacillus* | INI vs UNI0 | 9.85 × 10^-21^ | 3.94 × 10^-20^ |
| *Catenibacillus* | INI vs UNI25 | 3.34 × 10^-14^ | 3.34 × 10^-14^ |
| *Catenibacillus* | INI vs UNI50 | 2.95 × 10^-19^ | 5.9 × 10^-19^ |
| *Catenibacillus* | INI vs UNI75 | 1.56 × 10^-18^ | 2.08 × 10^-18^ |
| *Cetobacterium* | INI vs UNI0 | 9.85 × 10^-21^ | 3.94 × 10^-20^ |
| *Cetobacterium* | INI vs UNI25 | 3.34 × 10^-14^ | 3.34 × 10^-14^ |
| *Cetobacterium* | INI vs UNI50 | 2.95 × 10^-19^ | 5.9 × 10^-19^ |
| *Cetobacterium* | INI vs UNI75 | 1.56 × 10^-18^ | 2.08 × 10^-18^ |
| *Citrobacter* | INI vs UNI0 | 9.85 × 10^-21^ | 3.94 × 10^-20^ |
| *Citrobacter* | INI vs UNI25 | 3.34 × 10^-14^ | 3.34 × 10^-14^ |
| *Citrobacter* | INI vs UNI50 | 2.95 × 10^-19^ | 5.9 × 10^-19^ |
| *Citrobacter* | INI vs UNI75 | 1.56 × 10^-18^ | 2.08 × 10^-18^ |
| *Cloacibacterium* | INI vs UNI0 | 9.85 × 10^-21^ | 3.94 × 10^-20^ |
| *Cloacibacterium* | INI vs UNI25 | 3.34 × 10^-14^ | 3.34 × 10^-14^ |
| *Cloacibacterium* | INI vs UNI50 | 2.95 × 10^-19^ | 5.9 × 10^-19^ |
| *Cloacibacterium* | INI vs UNI75 | 1.56 × 10^-18^ | 2.08 × 10^-18^ |
| *Clostridium* | INI vs UNI0 | 9.85 × 10^-21^ | 3.94 × 10^-20^ |
| *Clostridium* | INI vs UNI25 | 3.34 × 10^-14^ | 3.34 × 10^-14^ |
| *Clostridium* | INI vs UNI50 | 2.95 × 10^-19^ | 5.9 × 10^-19^ |
| *Clostridium* | INI vs UNI75 | 1.56 × 10^-18^ | 2.08 × 10^-18^ |
| *Clostridium3* | INI vs UNI0 | 9.85 × 10^-21^ | 3.94 × 10^-20^ |
| *Clostridium3* | INI vs UNI25 | 3.34 × 10^-14^ | 3.34 × 10^-14^ |
| *Clostridium3* | INI vs UNI50 | 2.95 × 10^-19^ | 5.9 × 10^-19^ |
| *Clostridium3* | INI vs UNI75 | 1.56 × 10^-18^ | 2.08 × 10^-18^ |
| *Cohnella* | INI vs UNI0 | 9.85 × 10^-21^ | 3.94 × 10^-20^ |
| *Cohnella* | INI vs UNI25 | 3.34 × 10^-14^ | 3.34 × 10^-14^ |
| *Cohnella* | INI vs UNI50 | 2.95 × 10^-19^ | 5.9 × 10^-19^ |
| *Cohnella* | INI vs UNI75 | 1.56 × 10^-18^ | 2.08 × 10^-18^ |
| *UNI0exibacter* | INI vs UNI0 | 9.85 × 10^-21^ | 3.94 × 10^-20^ |
| *UNI0exibacter* | INI vs UNI25 | 3.34 × 10^-14^ | 3.34 × 10^-14^ |
| *UNI0exibacter* | INI vs UNI50 | 2.95 × 10^-19^ | 5.9 × 10^-19^ |
| *UNI0exibacter* | INI vs UNI75 | 1.56 × 10^-18^ | 2.08 × 10^-18^ |
| *Corynebacterium* | INI vs UNI0 | 9.85 × 10^-21^ | 3.94 × 10^-20^ |
| *Corynebacterium* | INI vs UNI25 | 3.34 × 10^-14^ | 3.34 × 10^-14^ |
| *Corynebacterium* | INI vs UNI50 | 2.95 × 10^-19^ | 5.9 × 10^-19^ |
| *Corynebacterium* | INI vs UNI75 | 1.56 × 10^-18^ | 2.08 × 10^-18^ |
| *Coxiella* | INI vs UNI0 | 9.85 × 10^-21^ | 3.94 × 10^-20^ |
| *Coxiella* | INI vs UNI25 | 3.34 × 10^-14^ | 3.34 × 10^-14^ |
| *Coxiella* | INI vs UNI50 | 2.95 × 10^-19^ | 5.9 × 10^-19^ |
| *Coxiella* | INI vs UNI75 | 1.56 × 10^-18^ | 2.08 × 10^-18^ |
| *Crenobacter* | INI vs UNI0 | 9.85 × 10^-21^ | 3.94 × 10^-20^ |
| *Crenobacter* | INI vs UNI25 | 3.34 × 10^-14^ | 3.34 × 10^-14^ |
| *Crenobacter* | INI vs UNI50 | 2.95 × 10^-19^ | 5.9 × 10^-19^ |
| *Crenobacter* | INI vs UNI75 | 1.56 × 10^-18^ | 2.08 × 10^-18^ |
| *Curtobacterium* | INI vs UNI0 | 9.85 × 10^-21^ | 3.94 × 10^-20^ |
| *Curtobacterium* | INI vs UNI25 | 3.34 × 10^-14^ | 3.34 × 10^-14^ |
| *Curtobacterium* | INI vs UNI50 | 2.95 × 10^-19^ | 5.9 × 10^-19^ |
| *Curtobacterium* | INI vs UNI75 | 1.56 × 10^-18^ | 2.08 × 10^-18^ |
| *Dermacoccus* | INI vs UNI0 | 9.85 × 10^-21^ | 3.94 × 10^-20^ |
| *Dermacoccus* | INI vs UNI25 | 3.34 × 10^-14^ | 3.34 × 10^-14^ |
| *Dermacoccus* | INI vs UNI50 | 2.95 × 10^-19^ | 5.9 × 10^-19^ |
| *Dermacoccus* | INI vs UNI75 | 1.56 × 10^-18^ | 2.08 × 10^-18^ |
| *Dysgonomonas* | INI vs UNI0 | 9.85 × 10^-21^ | 3.94 × 10^-20^ |
| *Dysgonomonas* | INI vs UNI25 | 3.34 × 10^-14^ | 3.34 × 10^-14^ |
| *Dysgonomonas* | INI vs UNI50 | 2.95 × 10^-19^ | 5.9 × 10^-19^ |
| *Dysgonomonas* | INI vs UNI75 | 1.56 × 10^-18^ | 2.08 × 10^-18^ |
| *Enhydrobacter* | INI vs UNI0 | 9.85 × 10^-21^ | 3.94 × 10^-20^ |
| *Enhydrobacter* | INI vs UNI25 | 3.34 × 10^-14^ | 3.34 × 10^-14^ |
| *Enhydrobacter* | INI vs UNI50 | 2.95 × 10^-19^ | 5.9 × 10^-19^ |
| *Enhydrobacter* | INI vs UNI75 | 1.56 × 10^-18^ | 2.08 × 10^-18^ |
| *Enterococcus* | INI vs UNI0 | 9.85 × 10^-21^ | 3.94 × 10^-20^ |
| *Enterococcus* | INI vs UNI25 | 3.34 × 10^-14^ | 3.34 × 10^-14^ |
| *Enterococcus* | INI vs UNI50 | 2.95 × 10^-19^ | 5.9 × 10^-19^ |
| *Enterococcus* | INI vs UNI75 | 1.56 × 10^-18^ | 2.08 × 10^-18^ |
| *Epulopiscium* | INI vs UNI0 | 9.85 × 10^-21^ | 3.94 × 10^-20^ |
| *Epulopiscium* | INI vs UNI25 | 3.34 × 10^-14^ | 3.34 × 10^-14^ |
| *Epulopiscium* | INI vs UNI50 | 2.95 × 10^-19^ | 5.9 × 10^-19^ |
| *Epulopiscium* | INI vs UNI75 | 1.56 × 10^-18^ | 2.08 × 10^-18^ |
| *Erysipelatoclostridium* | INI vs UNI0 | 9.85 × 10^-21^ | 3.94 × 10^-20^ |
| *Erysipelatoclostridium* | INI vs UNI25 | 3.34 × 10^-14^ | 3.34 × 10^-14^ |
| *Erysipelatoclostridium* | INI vs UNI50 | 2.95 × 10^-19^ | 5.9 × 10^-19^ |
| *Erysipelatoclostridium* | INI vs UNI75 | 1.56 × 10^-18^ | 2.08 × 10^-18^ |
| *Escherichia-Shigella* | INI vs UNI0 | 9.85 × 10^-21^ | 3.94 × 10^-20^ |
| *Escherichia-Shigella* | INI vs UNI25 | 3.34 × 10^-14^ | 3.34 × 10^-14^ |
| *Escherichia-Shigella* | INI vs UNI50 | 2.95 × 10^-19^ | 5.9 × 10^-19^ |
| *Escherichia-Shigella* | INI vs UNI75 | 1.56 × 10^-18^ | 2.08 × 10^-18^ |
| *Eubacterium* | INI vs UNI0 | 9.85 × 10^-21^ | 3.94 × 10^-20^ |
| *Eubacterium* | INI vs UNI25 | 3.34 × 10^-14^ | 3.34 × 10^-14^ |
| *Eubacterium* | INI vs UNI50 | 2.95 × 10^-19^ | 5.9 × 10^-19^ |
| *Eubacterium* | INI vs UNI75 | 1.56 × 10^-18^ | 2.08 × 10^-18^ |
| *Faecalitalea* | INI vs UNI0 | 9.85 × 10^-21^ | 3.94 × 10^-20^ |
| *Faecalitalea* | INI vs UNI25 | 3.34 × 10^-14^ | 3.34 × 10^-14^ |
| *Faecalitalea* | INI vs UNI50 | 2.95 × 10^-19^ | 5.9 × 10^-19^ |
| *Faecalitalea* | INI vs UNI75 | 1.56 × 10^-18^ | 2.08 × 10^-18^ |
| *Falsiporphyromonas* | INI vs UNI0 | 9.85 × 10^-21^ | 3.94 × 10^-20^ |
| *Falsiporphyromonas* | INI vs UNI25 | 3.34 × 10^-14^ | 3.34 × 10^-14^ |
| *Falsiporphyromonas* | INI vs UNI50 | 2.95 × 10^-19^ | 5.9 × 10^-19^ |
| *Falsiporphyromonas* | INI vs UNI75 | 1.56 × 10^-18^ | 2.08 × 10^-18^ |
| *Fictibacillus* | INI vs UNI0 | 9.85 × 10^-21^ | 3.94 × 10^-20^ |
| *Fictibacillus* | INI vs UNI25 | 3.34 × 10^-14^ | 3.34 × 10^-14^ |
| *Fictibacillus* | INI vs UNI50 | 2.95 × 10^-19^ | 5.9 × 10^-19^ |
| *Fictibacillus* | INI vs UNI75 | 1.56 × 10^-18^ | 2.08 × 10^-18^ |
| *Fimbriiglobus* | INI vs UNI0 | 9.85 × 10^-21^ | 3.94 × 10^-20^ |
| *Fimbriiglobus* | INI vs UNI25 | 3.34 × 10^-14^ | 3.34 × 10^-14^ |
| *Fimbriiglobus* | INI vs UNI50 | 2.95 × 10^-19^ | 5.9 × 10^-19^ |
| *Fimbriiglobus* | INI vs UNI75 | 1.56 × 10^-18^ | 2.08 × 10^-18^ |
| *Finegoldia* | INI vs UNI0 | 9.85 × 10^-21^ | 3.94 × 10^-20^ |
| *Finegoldia* | INI vs UNI25 | 3.34 × 10^-14^ | 3.34 × 10^-14^ |
| *Finegoldia* | INI vs UNI50 | 2.95 × 10^-19^ | 5.9 × 10^-19^ |
| *Finegoldia* | INI vs UNI75 | 1.56 × 10^-18^ | 2.08 × 10^-18^ |
| *Frisingicoccus* | INI vs UNI0 | 9.85 × 10^-21^ | 3.94 × 10^-20^ |
| *Frisingicoccus* | INI vs UNI25 | 3.34 × 10^-14^ | 3.34 × 10^-14^ |
| *Frisingicoccus* | INI vs UNI50 | 2.95 × 10^-19^ | 5.9 × 10^-19^ |
| *Frisingicoccus* | INI vs UNI75 | 1.56 × 10^-18^ | 2.08 × 10^-18^ |
| *Fusobacterium* | INI vs UNI0 | 9.85 × 10^-21^ | 3.94 × 10^-20^ |
| *Fusobacterium* | INI vs UNI25 | 3.34 × 10^-14^ | 3.34 × 10^-14^ |
| *Fusobacterium* | INI vs UNI50 | 2.95 × 10^-19^ | 5.9 × 10^-19^ |
| *Fusobacterium* | INI vs UNI75 | 1.56 × 10^-18^ | 2.08 × 10^-18^ |
| *Galbitalea* | INI vs UNI0 | 9.85 × 10^-21^ | 3.94 × 10^-20^ |
| *Galbitalea* | INI vs UNI25 | 3.34 × 10^-14^ | 3.34 × 10^-14^ |
| *Galbitalea* | INI vs UNI50 | 2.95 × 10^-19^ | 5.9 × 10^-19^ |
| *Galbitalea* | INI vs UNI75 | 1.56 × 10^-18^ | 2.08 × 10^-18^ |
| *Gallicola* | INI vs UNI0 | 9.85 × 10^-21^ | 3.94 × 10^-20^ |
| *Gallicola* | INI vs UNI25 | 3.34 × 10^-14^ | 3.34 × 10^-14^ |
| *Gallicola* | INI vs UNI50 | 2.95 × 10^-19^ | 5.9 × 10^-19^ |
| *Gallicola* | INI vs UNI75 | 1.56 × 10^-18^ | 2.08 × 10^-18^ |
| *Gemmobacter* | INI vs UNI0 | 9.85 × 10^-21^ | 3.94 × 10^-20^ |
| *Gemmobacter* | INI vs UNI25 | 3.34 × 10^-14^ | 3.34 × 10^-14^ |
| *Gemmobacter* | INI vs UNI50 | 2.95 × 10^-19^ | 5.9 × 10^-19^ |
| *Gemmobacter* | INI vs UNI75 | 1.56 × 10^-18^ | 2.08 × 10^-18^ |
| *Geobacillus* | INI vs UNI0 | 9.85 × 10^-21^ | 3.94 × 10^-20^ |
| *Geobacillus* | INI vs UNI25 | 3.34 × 10^-14^ | 3.34 × 10^-14^ |
| *Geobacillus* | INI vs UNI50 | 2.95 × 10^-19^ | 5.9 × 10^-19^ |
| *Geobacillus* | INI vs UNI75 | 1.56 × 10^-18^ | 2.08 × 10^-18^ |
| *Glutamicibacter* | INI vs UNI0 | 9.85 × 10^-21^ | 3.94 × 10^-20^ |
| *Glutamicibacter* | INI vs UNI25 | 3.34 × 10^-14^ | 3.34 × 10^-14^ |
| *Glutamicibacter* | INI vs UNI50 | 2.95 × 10^-19^ | 5.9 × 10^-19^ |
| *Glutamicibacter* | INI vs UNI75 | 1.56 × 10^-18^ | 2.08 × 10^-18^ |
| *Gordonia* | INI vs UNI0 | 9.85 × 10^-21^ | 3.94 × 10^-20^ |
| *Gordonia* | INI vs UNI25 | 3.34 × 10^-14^ | 3.34 × 10^-14^ |
| *Gordonia* | INI vs UNI50 | 2.95 × 10^-19^ | 5.9 × 10^-19^ |
| *Gordonia* | INI vs UNI75 | 1.56 × 10^-18^ | 2.08 × 10^-18^ |
| *Granulicatella* | INI vs UNI0 | 9.85 × 10^-21^ | 3.94 × 10^-20^ |
| *Granulicatella* | INI vs UNI25 | 3.34 × 10^-14^ | 3.34 × 10^-14^ |
| *Granulicatella* | INI vs UNI50 | 2.95 × 10^-19^ | 5.9 × 10^-19^ |
| *Granulicatella* | INI vs UNI75 | 1.56 × 10^-18^ | 2.08 × 10^-18^ |
| *HT002* | INI vs UNI0 | 9.85 × 10^-21^ | 3.94 × 10^-20^ |
| *HT002* | INI vs UNI25 | 3.34 × 10^-14^ | 3.34 × 10^-14^ |
| *HT002* | INI vs UNI50 | 2.95 × 10^-19^ | 5.9 × 10^-19^ |
| *HT002* | INI vs UNI75 | 1.56 × 10^-18^ | 2.08 × 10^-18^ |
| *Haloimpatiens* | INI vs UNI0 | 9.85 × 10^-21^ | 3.94 × 10^-20^ |
| *Haloimpatiens* | INI vs UNI25 | 3.34 × 10^-14^ | 3.34 × 10^-14^ |
| *Haloimpatiens* | INI vs UNI50 | 2.95 × 10^-19^ | 5.9 × 10^-19^ |
| *Haloimpatiens* | INI vs UNI75 | 1.56 × 10^-18^ | 2.08 × 10^-18^ |
| *Hathewaya* | INI vs UNI0 | 9.85 × 10^-21^ | 3.94 × 10^-20^ |
| *Hathewaya* | INI vs UNI25 | 3.34 × 10^-14^ | 3.34 × 10^-14^ |
| *Hathewaya* | INI vs UNI50 | 2.95 × 10^-19^ | 5.9 × 10^-19^ |
| *Hathewaya* | INI vs UNI75 | 1.56 × 10^-18^ | 2.08 × 10^-18^ |
| *Helcococcus* | INI vs UNI0 | 9.85 × 10^-21^ | 3.94 × 10^-20^ |
| *Helcococcus* | INI vs UNI25 | 3.34 × 10^-14^ | 3.34 × 10^-14^ |
| *Helcococcus* | INI vs UNI50 | 2.95 × 10^-19^ | 5.9 × 10^-19^ |
| *Helcococcus* | INI vs UNI75 | 1.56 × 10^-18^ | 2.08 × 10^-18^ |
| *Hespellia* | INI vs UNI0 | 9.85 × 10^-21^ | 3.94 × 10^-20^ |
| *Hespellia* | INI vs UNI25 | 3.34 × 10^-14^ | 3.34 × 10^-14^ |
| *Hespellia* | INI vs UNI50 | 2.95 × 10^-19^ | 5.9 × 10^-19^ |
| *Hespellia* | INI vs UNI75 | 1.56 × 10^-18^ | 2.08 × 10^-18^ |
| *Hyphomicrobium* | INI vs UNI0 | 9.85 × 10^-21^ | 3.94 × 10^-20^ |
| *Hyphomicrobium* | INI vs UNI25 | 3.34 × 10^-14^ | 3.34 × 10^-14^ |
| *Hyphomicrobium* | INI vs UNI50 | 2.95 × 10^-19^ | 5.9 × 10^-19^ |
| *Hyphomicrobium* | INI vs UNI75 | 1.56 × 10^-18^ | 2.08 × 10^-18^ |
| *IMCC26207* | INI vs UNI0 | 9.85 × 10^-21^ | 3.94 × 10^-20^ |
| *IMCC26207* | INI vs UNI25 | 3.34 × 10^-14^ | 3.34 × 10^-14^ |
| *IMCC26207* | INI vs UNI50 | 2.95 × 10^-19^ | 5.9 × 10^-19^ |
| *IMCC26207* | INI vs UNI75 | 1.56 × 10^-18^ | 2.08 × 10^-18^ |
| *Iamia* | INI vs UNI0 | 9.85 × 10^-21^ | 3.94 × 10^-20^ |
| *Iamia* | INI vs UNI25 | 3.34 × 10^-14^ | 3.34 × 10^-14^ |
| *Iamia* | INI vs UNI50 | 2.95 × 10^-19^ | 5.9 × 10^-19^ |
| *Iamia* | INI vs UNI75 | 1.56 × 10^-18^ | 2.08 × 10^-18^ |
| *Ignatzschineria* | INI vs UNI0 | 9.85 × 10^-21^ | 3.94 × 10^-20^ |
| *Ignatzschineria* | INI vs UNI25 | 3.34 × 10^-14^ | 3.34 × 10^-14^ |
| *Ignatzschineria* | INI vs UNI50 | 2.95 × 10^-19^ | 5.9 × 10^-19^ |
| *Ignatzschineria* | INI vs UNI75 | 1.56 × 10^-18^ | 2.08 × 10^-18^ |
| *Lacticaseibacillus* | INI vs UNI0 | 9.85 × 10^-21^ | 3.94 × 10^-20^ |
| *Lacticaseibacillus* | INI vs UNI25 | 3.34 × 10^-14^ | 3.34 × 10^-14^ |
| *Lacticaseibacillus* | INI vs UNI50 | 2.95 × 10^-19^ | 5.9 × 10^-19^ |
| *Lacticaseibacillus* | INI vs UNI75 | 1.56 × 10^-18^ | 2.08 × 10^-18^ |
| *Lactiplantibacillus* | INI vs UNI0 | 9.85 × 10^-21^ | 3.94 × 10^-20^ |
| *Lactiplantibacillus* | INI vs UNI25 | 3.34 × 10^-14^ | 3.34 × 10^-14^ |
| *Lactiplantibacillus* | INI vs UNI50 | 2.95 × 10^-19^ | 5.9 × 10^-19^ |
| *Lactiplantibacillus* | INI vs UNI75 | 1.56 × 10^-18^ | 2.08 × 10^-18^ |
| *Lactobacillus* | INI vs UNI0 | 9.85 × 10^-21^ | 3.94 × 10^-20^ |
| *Lactobacillus* | INI vs UNI25 | 3.34 × 10^-14^ | 3.34 × 10^-14^ |
| *Lactobacillus* | INI vs UNI50 | 2.95 × 10^-19^ | 5.9 × 10^-19^ |
| *Lactobacillus* | INI vs UNI75 | 1.56 × 10^-18^ | 2.08 × 10^-18^ |
| *Lactococcus* | INI vs UNI0 | 9.85 × 10^-21^ | 3.94 × 10^-20^ |
| *Lactococcus* | INI vs UNI25 | 3.34 × 10^-14^ | 3.34 × 10^-14^ |
| *Lactococcus* | INI vs UNI50 | 2.95 × 10^-19^ | 5.9 × 10^-19^ |
| *Lactococcus* | INI vs UNI75 | 1.56 × 10^-18^ | 2.08 × 10^-18^ |
| *Latilactobacillus* | INI vs UNI0 | 9.85 × 10^-21^ | 3.94 × 10^-20^ |
| *Latilactobacillus* | INI vs UNI25 | 3.34 × 10^-14^ | 3.34 × 10^-14^ |
| *Latilactobacillus* | INI vs UNI50 | 2.95 × 10^-19^ | 5.9 × 10^-19^ |
| *Latilactobacillus* | INI vs UNI75 | 1.56 × 10^-18^ | 2.08 × 10^-18^ |
| *Legionella* | INI vs UNI0 | 9.85 × 10^-21^ | 3.94 × 10^-20^ |
| *Legionella* | INI vs UNI25 | 3.34 × 10^-14^ | 3.34 × 10^-14^ |
| *Legionella* | INI vs UNI50 | 2.95 × 10^-19^ | 5.9 × 10^-19^ |
| *Legionella* | INI vs UNI75 | 1.56 × 10^-18^ | 2.08 × 10^-18^ |
| *Lentilactobacillus* | INI vs UNI0 | 9.85 × 10^-21^ | 3.94 × 10^-20^ |
| *Lentilactobacillus* | INI vs UNI25 | 3.34 × 10^-14^ | 3.34 × 10^-14^ |
| *Lentilactobacillus* | INI vs UNI50 | 2.95 × 10^-19^ | 5.9 × 10^-19^ |
| *Lentilactobacillus* | INI vs UNI75 | 1.56 × 10^-18^ | 2.08 × 10^-18^ |
| *Leucobacter* | INI vs UNI0 | 9.85 × 10^-21^ | 3.94 × 10^-20^ |
| *Leucobacter* | INI vs UNI25 | 3.34 × 10^-14^ | 3.34 × 10^-14^ |
| *Leucobacter* | INI vs UNI50 | 2.95 × 10^-19^ | 5.9 × 10^-19^ |
| *Leucobacter* | INI vs UNI75 | 1.56 × 10^-18^ | 2.08 × 10^-18^ |
| *LeuUNI0ostoc* | INI vs UNI0 | 9.85 × 10^-21^ | 3.94 × 10^-20^ |
| *LeuUNI0ostoc* | INI vs UNI25 | 3.34 × 10^-14^ | 3.34 × 10^-14^ |
| *LeuUNI0ostoc* | INI vs UNI50 | 2.95 × 10^-19^ | 5.9 × 10^-19^ |
| *LeuUNI0ostoc* | INI vs UNI75 | 1.56 × 10^-18^ | 2.08 × 10^-18^ |
| *Ligilactobacillus* | INI vs UNI0 | 9.85 × 10^-21^ | 3.94 × 10^-20^ |
| *Ligilactobacillus* | INI vs UNI25 | 3.34 × 10^-14^ | 3.34 × 10^-14^ |
| *Ligilactobacillus* | INI vs UNI50 | 2.95 × 10^-19^ | 5.9 × 10^-19^ |
| *Ligilactobacillus* | INI vs UNI75 | 1.56 × 10^-18^ | 2.08 × 10^-18^ |
| *Limosilactobacillus* | INI vs UNI0 | 9.85 × 10^-21^ | 3.94 × 10^-20^ |
| *Limosilactobacillus* | INI vs UNI25 | 3.34 × 10^-14^ | 3.34 × 10^-14^ |
| *Limosilactobacillus* | INI vs UNI50 | 2.95 × 10^-19^ | 5.9 × 10^-19^ |
| *Limosilactobacillus* | INI vs UNI75 | 1.56 × 10^-18^ | 2.08 × 10^-18^ |
| *Listeria* | INI vs UNI0 | 9.85 × 10^-21^ | 3.94 × 10^-20^ |
| *Listeria* | INI vs UNI25 | 3.34 × 10^-14^ | 3.34 × 10^-14^ |
| *Listeria* | INI vs UNI50 | 2.95 × 10^-19^ | 5.9 × 10^-19^ |
| *Listeria* | INI vs UNI75 | 1.56 × 10^-18^ | 2.08 × 10^-18^ |
| *Longivirga* | INI vs UNI0 | 9.85 × 10^-21^ | 3.94 × 10^-20^ |
| *Longivirga* | INI vs UNI25 | 3.34 × 10^-14^ | 3.34 × 10^-14^ |
| *Longivirga* | INI vs UNI50 | 2.95 × 10^-19^ | 5.9 × 10^-19^ |
| *Longivirga* | INI vs UNI75 | 1.56 × 10^-18^ | 2.08 × 10^-18^ |
| *Lysinibacillus* | INI vs UNI0 | 9.85 × 10^-21^ | 3.94 × 10^-20^ |
| *Lysinibacillus* | INI vs UNI25 | 3.34 × 10^-14^ | 3.34 × 10^-14^ |
| *Lysinibacillus* | INI vs UNI50 | 2.95 × 10^-19^ | 5.9 × 10^-19^ |
| *Lysinibacillus* | INI vs UNI75 | 1.56 × 10^-18^ | 2.08 × 10^-18^ |
| *Megasphaera* | INI vs UNI0 | 9.85 × 10^-21^ | 3.94 × 10^-20^ |
| *Megasphaera* | INI vs UNI25 | 3.34 × 10^-14^ | 3.34 × 10^-14^ |
| *Megasphaera* | INI vs UNI50 | 2.95 × 10^-19^ | 5.9 × 10^-19^ |
| *Megasphaera* | INI vs UNI75 | 1.56 × 10^-18^ | 2.08 × 10^-18^ |
| *Methylococcus* | INI vs UNI0 | 9.85 × 10^-21^ | 3.94 × 10^-20^ |
| *Methylococcus* | INI vs UNI25 | 3.34 × 10^-14^ | 3.34 × 10^-14^ |
| *Methylococcus* | INI vs UNI50 | 2.95 × 10^-19^ | 5.9 × 10^-19^ |
| *Methylococcus* | INI vs UNI75 | 1.56 × 10^-18^ | 2.08 × 10^-18^ |
| *Methylocystis* | INI vs UNI0 | 9.85 × 10^-21^ | 3.94 × 10^-20^ |
| *Methylocystis* | INI vs UNI25 | 3.34 × 10^-14^ | 3.34 × 10^-14^ |
| *Methylocystis* | INI vs UNI50 | 2.95 × 10^-19^ | 5.9 × 10^-19^ |
| *Methylocystis* | INI vs UNI75 | 1.56 × 10^-18^ | 2.08 × 10^-18^ |
| *Methylophilus* | INI vs UNI0 | 9.85 × 10^-21^ | 3.94 × 10^-20^ |
| *Methylophilus* | INI vs UNI25 | 3.34 × 10^-14^ | 3.34 × 10^-14^ |
| *Methylophilus* | INI vs UNI50 | 2.95 × 10^-19^ | 5.9 × 10^-19^ |
| *Methylophilus* | INI vs UNI75 | 1.56 × 10^-18^ | 2.08 × 10^-18^ |
| *Minicystis* | INI vs UNI0 | 9.85 × 10^-21^ | 3.94 × 10^-20^ |
| *Minicystis* | INI vs UNI25 | 3.34 × 10^-14^ | 3.34 × 10^-14^ |
| *Minicystis* | INI vs UNI50 | 2.95 × 10^-19^ | 5.9 × 10^-19^ |
| *Minicystis* | INI vs UNI75 | 1.56 × 10^-18^ | 2.08 × 10^-18^ |
| *Mycobacterium* | INI vs UNI0 | 9.85 × 10^-21^ | 3.94 × 10^-20^ |
| *Mycobacterium* | INI vs UNI25 | 3.34 × 10^-14^ | 3.34 × 10^-14^ |
| *Mycobacterium* | INI vs UNI50 | 2.95 × 10^-19^ | 5.9 × 10^-19^ |
| *Mycobacterium* | INI vs UNI75 | 1.56 × 10^-18^ | 2.08 × 10^-18^ |
| *Myroides* | INI vs UNI0 | 9.85 × 10^-21^ | 3.94 × 10^-20^ |
| *Myroides* | INI vs UNI25 | 3.34 × 10^-14^ | 3.34 × 10^-14^ |
| *Myroides* | INI vs UNI50 | 2.95 × 10^-19^ | 5.9 × 10^-19^ |
| *Myroides* | INI vs UNI75 | 1.56 × 10^-18^ | 2.08 × 10^-18^ |
| *Nocardia* | INI vs UNI0 | 9.85 × 10^-21^ | 3.94 × 10^-20^ |
| *Nocardia* | INI vs UNI25 | 3.34 × 10^-14^ | 3.34 × 10^-14^ |
| *Nocardia* | INI vs UNI50 | 2.95 × 10^-19^ | 5.9 × 10^-19^ |
| *Nocardia* | INI vs UNI75 | 1.56 × 10^-18^ | 2.08 × 10^-18^ |
| *Oceanobacillus* | INI vs UNI0 | 9.85 × 10^-21^ | 3.94 × 10^-20^ |
| *Oceanobacillus* | INI vs UNI25 | 3.34 × 10^-14^ | 3.34 × 10^-14^ |
| *Oceanobacillus* | INI vs UNI50 | 2.95 × 10^-19^ | 5.9 × 10^-19^ |
| *Oceanobacillus* | INI vs UNI75 | 1.56 × 10^-18^ | 2.08 × 10^-18^ |
| *Others* | INI vs UNI0 | 9.85 × 10^-21^ | 3.94 × 10^-20^ |
| *Others* | INI vs UNI25 | 3.34 × 10^-14^ | 3.34 × 10^-14^ |
| *Others* | INI vs UNI50 | 2.95 × 10^-19^ | 5.9 × 10^-19^ |
| *Others* | INI vs UNI75 | 1.56 × 10^-18^ | 2.08 × 10^-18^ |
| *Paenibacillus* | INI vs UNI0 | 9.85 × 10^-21^ | 3.94 × 10^-20^ |
| *Paenibacillus* | INI vs UNI25 | 3.34 × 10^-14^ | 3.34 × 10^-14^ |
| *Paenibacillus* | INI vs UNI50 | 2.95 × 10^-19^ | 5.9 × 10^-19^ |
| *Paenibacillus* | INI vs UNI75 | 1.56 × 10^-18^ | 2.08 × 10^-18^ |
| *Paeniclostridium* | INI vs UNI0 | 9.85 × 10^-21^ | 3.94 × 10^-20^ |
| *Paeniclostridium* | INI vs UNI25 | 3.34 × 10^-14^ | 3.34 × 10^-14^ |
| *Paeniclostridium* | INI vs UNI50 | 2.95 × 10^-19^ | 5.9 × 10^-19^ |
| *Paeniclostridium* | INI vs UNI75 | 1.56 × 10^-18^ | 2.08 × 10^-18^ |
| *Paraclostridium* | INI vs UNI0 | 9.85 × 10^-21^ | 3.94 × 10^-20^ |
| *Paraclostridium* | INI vs UNI25 | 3.34 × 10^-14^ | 3.34 × 10^-14^ |
| *Paraclostridium* | INI vs UNI50 | 2.95 × 10^-19^ | 5.9 × 10^-19^ |
| *Paraclostridium* | INI vs UNI75 | 1.56 × 10^-18^ | 2.08 × 10^-18^ |
| *Paucilactobacillus* | INI vs UNI0 | 9.85 × 10^-21^ | 3.94 × 10^-20^ |
| *Paucilactobacillus* | INI vs UNI25 | 3.34 × 10^-14^ | 3.34 × 10^-14^ |
| *Paucilactobacillus* | INI vs UNI50 | 2.95 × 10^-19^ | 5.9 × 10^-19^ |
| *Paucilactobacillus* | INI vs UNI75 | 1.56 × 10^-18^ | 2.08 × 10^-18^ |
| *Pediococcus* | INI vs UNI0 | 9.85 × 10^-21^ | 3.94 × 10^-20^ |
| *Pediococcus* | INI vs UNI25 | 3.34 × 10^-14^ | 3.34 × 10^-14^ |
| *Pediococcus* | INI vs UNI50 | 2.95 × 10^-19^ | 5.9 × 10^-19^ |
| *Pediococcus* | INI vs UNI75 | 1.56 × 10^-18^ | 2.08 × 10^-18^ |
| *Peptococcus* | INI vs UNI0 | 9.85 × 10^-21^ | 3.94 × 10^-20^ |
| *Peptococcus* | INI vs UNI25 | 3.34 × 10^-14^ | 3.34 × 10^-14^ |
| *Peptococcus* | INI vs UNI50 | 2.95 × 10^-19^ | 5.9 × 10^-19^ |
| *Peptococcus* | INI vs UNI75 | 1.56 × 10^-18^ | 2.08 × 10^-18^ |
| *Peptoniphilus* | INI vs UNI0 | 9.85 × 10^-21^ | 3.94 × 10^-20^ |
| *Peptoniphilus* | INI vs UNI25 | 3.34 × 10^-14^ | 3.34 × 10^-14^ |
| *Peptoniphilus* | INI vs UNI50 | 2.95 × 10^-19^ | 5.9 × 10^-19^ |
| *Peptoniphilus* | INI vs UNI75 | 1.56 × 10^-18^ | 2.08 × 10^-18^ |
| *Peptostreptococcus* | INI vs UNI0 | 9.85 × 10^-21^ | 3.94 × 10^-20^ |
| *Peptostreptococcus* | INI vs UNI25 | 3.34 × 10^-14^ | 3.34 × 10^-14^ |
| *Peptostreptococcus* | INI vs UNI50 | 2.95 × 10^-19^ | 5.9 × 10^-19^ |
| *Peptostreptococcus* | INI vs UNI75 | 1.56 × 10^-18^ | 2.08 × 10^-18^ |
| *Photobacterium* | INI vs UNI0 | 9.85 × 10^-21^ | 3.94 × 10^-20^ |
| *Photobacterium* | INI vs UNI25 | 3.34 × 10^-14^ | 3.34 × 10^-14^ |
| *Photobacterium* | INI vs UNI50 | 2.95 × 10^-19^ | 5.9 × 10^-19^ |
| *Photobacterium* | INI vs UNI75 | 1.56 × 10^-18^ | 2.08 × 10^-18^ |
| *Phreatobacter* | INI vs UNI0 | 9.85 × 10^-21^ | 3.94 × 10^-20^ |
| *Phreatobacter* | INI vs UNI25 | 3.34 × 10^-14^ | 3.34 × 10^-14^ |
| *Phreatobacter* | INI vs UNI50 | 2.95 × 10^-19^ | 5.9 × 10^-19^ |
| *Phreatobacter* | INI vs UNI75 | 1.56 × 10^-18^ | 2.08 × 10^-18^ |
| *Plesiomonas* | INI vs UNI0 | 9.85 × 10^-21^ | 3.94 × 10^-20^ |
| *Plesiomonas* | INI vs UNI25 | 3.34 × 10^-14^ | 3.34 × 10^-14^ |
| *Plesiomonas* | INI vs UNI50 | 2.95 × 10^-19^ | 5.9 × 10^-19^ |
| *Plesiomonas* | INI vs UNI75 | 1.56 × 10^-18^ | 2.08 × 10^-18^ |
| *Polynucleobacter* | INI vs UNI0 | 9.85 × 10^-21^ | 3.94 × 10^-20^ |
| *Polynucleobacter* | INI vs UNI25 | 3.34 × 10^-14^ | 3.34 × 10^-14^ |
| *Polynucleobacter* | INI vs UNI50 | 2.95 × 10^-19^ | 5.9 × 10^-19^ |
| *Polynucleobacter* | INI vs UNI75 | 1.56 × 10^-18^ | 2.08 × 10^-18^ |
| *Porphyromonas* | INI vs UNI0 | 9.85 × 10^-21^ | 3.94 × 10^-20^ |
| *Porphyromonas* | INI vs UNI25 | 3.34 × 10^-14^ | 3.34 × 10^-14^ |
| *Porphyromonas* | INI vs UNI50 | 2.95 × 10^-19^ | 5.9 × 10^-19^ |
| *Porphyromonas* | INI vs UNI75 | 1.56 × 10^-18^ | 2.08 × 10^-18^ |
| *Proteiniclasticum* | INI vs UNI0 | 9.85 × 10^-21^ | 3.94 × 10^-20^ |
| *Proteiniclasticum* | INI vs UNI25 | 3.34 × 10^-14^ | 3.34 × 10^-14^ |
| *Proteiniclasticum* | INI vs UNI50 | 2.95 × 10^-19^ | 5.9 × 10^-19^ |
| *Proteiniclasticum* | INI vs UNI75 | 1.56 × 10^-18^ | 2.08 × 10^-18^ |
| *Proteus* | INI vs UNI0 | 9.85 × 10^-21^ | 3.94 × 10^-20^ |
| *Proteus* | INI vs UNI25 | 3.34 × 10^-14^ | 3.34 × 10^-14^ |
| *Proteus* | INI vs UNI50 | 2.95 × 10^-19^ | 5.9 × 10^-19^ |
| *Proteus* | INI vs UNI75 | 1.56 × 10^-18^ | 2.08 × 10^-18^ |
| *Pseudomonas* | INI vs UNI0 | 9.85 × 10^-21^ | 3.94 × 10^-20^ |
| *Pseudomonas* | INI vs UNI25 | 3.34 × 10^-14^ | 3.34 × 10^-14^ |
| *Pseudomonas* | INI vs UNI50 | 2.95 × 10^-19^ | 5.9 × 10^-19^ |
| *Pseudomonas* | INI vs UNI75 | 1.56 × 10^-18^ | 2.08 × 10^-18^ |
| *Pseudonocardia* | INI vs UNI0 | 9.85 × 10^-21^ | 3.94 × 10^-20^ |
| *Pseudonocardia* | INI vs UNI25 | 3.34 × 10^-14^ | 3.34 × 10^-14^ |
| *Pseudonocardia* | INI vs UNI50 | 2.95 × 10^-19^ | 5.9 × 10^-19^ |
| *Pseudonocardia* | INI vs UNI75 | 1.56 × 10^-18^ | 2.08 × 10^-18^ |
| *Pseudoramibacter* | INI vs UNI0 | 9.85 × 10^-21^ | 3.94 × 10^-20^ |
| *Pseudoramibacter* | INI vs UNI25 | 3.34 × 10^-14^ | 3.34 × 10^-14^ |
| *Pseudoramibacter* | INI vs UNI50 | 2.95 × 10^-19^ | 5.9 × 10^-19^ |
| *Pseudoramibacter* | INI vs UNI75 | 1.56 × 10^-18^ | 2.08 × 10^-18^ |
| *Reyranella* | INI vs UNI0 | 9.85 × 10^-21^ | 3.94 × 10^-20^ |
| *Reyranella* | INI vs UNI25 | 3.34 × 10^-14^ | 3.34 × 10^-14^ |
| *Reyranella* | INI vs UNI50 | 2.95 × 10^-19^ | 5.9 × 10^-19^ |
| *Reyranella* | INI vs UNI75 | 1.56 × 10^-18^ | 2.08 × 10^-18^ |
| *Rhodobacter* | INI vs UNI0 | 9.85 × 10^-21^ | 3.94 × 10^-20^ |
| *Rhodobacter* | INI vs UNI25 | 3.34 × 10^-14^ | 3.34 × 10^-14^ |
| *Rhodobacter* | INI vs UNI50 | 2.95 × 10^-19^ | 5.9 × 10^-19^ |
| *Rhodobacter* | INI vs UNI75 | 1.56 × 10^-18^ | 2.08 × 10^-18^ |
| *Rhodococcus* | INI vs UNI0 | 9.85 × 10^-21^ | 3.94 × 10^-20^ |
| *Rhodococcus* | INI vs UNI25 | 3.34 × 10^-14^ | 3.34 × 10^-14^ |
| *Rhodococcus* | INI vs UNI50 | 2.95 × 10^-19^ | 5.9 × 10^-19^ |
| *Rhodococcus* | INI vs UNI75 | 1.56 × 10^-18^ | 2.08 × 10^-18^ |
| *Romboutsia* | INI vs UNI0 | 9.85 × 10^-21^ | 3.94 × 10^-20^ |
| *Romboutsia* | INI vs UNI25 | 3.34 × 10^-14^ | 3.34 × 10^-14^ |
| *Romboutsia* | INI vs UNI50 | 2.95 × 10^-19^ | 5.9 × 10^-19^ |
| *Romboutsia* | INI vs UNI75 | 1.56 × 10^-18^ | 2.08 × 10^-18^ |
| *SH-PL14* | INI vs UNI0 | 9.85 × 10^-21^ | 3.94 × 10^-20^ |
| *SH-PL14* | INI vs UNI25 | 3.34 × 10^-14^ | 3.34 × 10^-14^ |
| *SH-PL14* | INI vs UNI50 | 2.95 × 10^-19^ | 5.9 × 10^-19^ |
| *SH-PL14* | INI vs UNI75 | 1.56 × 10^-18^ | 2.08 × 10^-18^ |
| *Salmonella* | INI vs UNI0 | 9.85 × 10^-21^ | 3.94 × 10^-20^ |
| *Salmonella* | INI vs UNI25 | 3.34 × 10^-14^ | 3.34 × 10^-14^ |
| *Salmonella* | INI vs UNI50 | 2.95 × 10^-19^ | 5.9 × 10^-19^ |
| *Salmonella* | INI vs UNI75 | 1.56 × 10^-18^ | 2.08 × 10^-18^ |
| *Shewanella* | INI vs UNI0 | 9.85 × 10^-21^ | 3.94 × 10^-20^ |
| *Shewanella* | INI vs UNI25 | 3.34 × 10^-14^ | 3.34 × 10^-14^ |
| *Shewanella* | INI vs UNI50 | 2.95 × 10^-19^ | 5.9 × 10^-19^ |
| *Shewanella* | INI vs UNI75 | 1.56 × 10^-18^ | 2.08 × 10^-18^ |
| *Sphingomonas* | INI vs UNI0 | 9.85 × 10^-21^ | 3.94 × 10^-20^ |
| *Sphingomonas* | INI vs UNI25 | 3.34 × 10^-14^ | 3.34 × 10^-14^ |
| *Sphingomonas* | INI vs UNI50 | 2.95 × 10^-19^ | 5.9 × 10^-19^ |
| *Sphingomonas* | INI vs UNI75 | 1.56 × 10^-18^ | 2.08 × 10^-18^ |
| *Staphylococcus* | INI vs UNI0 | 9.85 × 10^-21^ | 3.94 × 10^-20^ |
| *Staphylococcus* | INI vs UNI25 | 3.34 × 10^-14^ | 3.34 × 10^-14^ |
| *Staphylococcus* | INI vs UNI50 | 2.95 × 10^-19^ | 5.9 × 10^-19^ |
| *Staphylococcus* | INI vs UNI75 | 1.56 × 10^-18^ | 2.08 × 10^-18^ |
| *Stenotrophomonas* | INI vs UNI0 | 9.85 × 10^-21^ | 3.94 × 10^-20^ |
| *Stenotrophomonas* | INI vs UNI25 | 3.34 × 10^-14^ | 3.34 × 10^-14^ |
| *Stenotrophomonas* | INI vs UNI50 | 2.95 × 10^-19^ | 5.9 × 10^-19^ |
| *Stenotrophomonas* | INI vs UNI75 | 1.56 × 10^-18^ | 2.08 × 10^-18^ |
| *Streptococcus* | INI vs UNI0 | 9.85 × 10^-21^ | 3.94 × 10^-20^ |
| *Streptococcus* | INI vs UNI25 | 3.34 × 10^-14^ | 3.34 × 10^-14^ |
| *Streptococcus* | INI vs UNI50 | 2.95 × 10^-19^ | 5.9 × 10^-19^ |
| *Streptococcus* | INI vs UNI75 | 1.56 × 10^-18^ | 2.08 × 10^-18^ |
| *Tabrizicola* | INI vs UNI0 | 9.85 × 10^-21^ | 3.94 × 10^-20^ |
| *Tabrizicola* | INI vs UNI25 | 3.34 × 10^-14^ | 3.34 × 10^-14^ |
| *Tabrizicola* | INI vs UNI50 | 2.95 × 10^-19^ | 5.9 × 10^-19^ |
| *Tabrizicola* | INI vs UNI75 | 1.56 × 10^-18^ | 2.08 × 10^-18^ |
| *Tepidimonas* | INI vs UNI0 | 9.85 × 10^-21^ | 3.94 × 10^-20^ |
| *Tepidimonas* | INI vs UNI25 | 3.34 × 10^-14^ | 3.34 × 10^-14^ |
| *Tepidimonas* | INI vs UNI50 | 2.95 × 10^-19^ | 5.9 × 10^-19^ |
| *Tepidimonas* | INI vs UNI75 | 1.56 × 10^-18^ | 2.08 × 10^-18^ |
| *Terrimicrobium* | INI vs UNI0 | 9.85 × 10^-21^ | 3.94 × 10^-20^ |
| *Terrimicrobium* | INI vs UNI25 | 3.34 × 10^-14^ | 3.34 × 10^-14^ |
| *Terrimicrobium* | INI vs UNI50 | 2.95 × 10^-19^ | 5.9 × 10^-19^ |
| *Terrimicrobium* | INI vs UNI75 | 1.56 × 10^-18^ | 2.08 × 10^-18^ |
| *Terrisporobacter* | INI vs UNI0 | 9.85 × 10^-21^ | 3.94 × 10^-20^ |
| *Terrisporobacter* | INI vs UNI25 | 3.34 × 10^-14^ | 3.34 × 10^-14^ |
| *Terrisporobacter* | INI vs UNI50 | 2.95 × 10^-19^ | 5.9 × 10^-19^ |
| *Terrisporobacter* | INI vs UNI75 | 1.56 × 10^-18^ | 2.08 × 10^-18^ |
| *Trichococcus* | INI vs UNI0 | 9.85 × 10^-21^ | 3.94 × 10^-20^ |
| *Trichococcus* | INI vs UNI25 | 3.34 × 10^-14^ | 3.34 × 10^-14^ |
| *Trichococcus* | INI vs UNI50 | 2.95 × 10^-19^ | 5.9 × 10^-19^ |
| *Trichococcus* | INI vs UNI75 | 1.56 × 10^-18^ | 2.08 × 10^-18^ |
| *Turicibacter* | INI vs UNI0 | 9.85 × 10^-21^ | 3.94 × 10^-20^ |
| *Turicibacter* | INI vs UNI25 | 3.34 × 10^-14^ | 3.34 × 10^-14^ |
| *Turicibacter* | INI vs UNI50 | 2.95 × 10^-19^ | 5.9 × 10^-19^ |
| *Turicibacter* | INI vs UNI75 | 1.56 × 10^-18^ | 2.08 × 10^-18^ |
| *UCG-008* | INI vs UNI0 | 9.85 × 10^-21^ | 3.94 × 10^-20^ |
| *UCG-008* | INI vs UNI25 | 3.34 × 10^-14^ | 3.34 × 10^-14^ |
| *UCG-008* | INI vs UNI50 | 2.95 × 10^-19^ | 5.9 × 10^-19^ |
| *UCG-008* | INI vs UNI75 | 1.56 × 10^-18^ | 2.08 × 10^-18^ |
| *Vagococcus* | INI vs UNI0 | 9.85 × 10^-21^ | 3.94 × 10^-20^ |
| *Vagococcus* | INI vs UNI25 | 3.34 × 10^-14^ | 3.34 × 10^-14^ |
| *Vagococcus* | INI vs UNI50 | 2.95 × 10^-19^ | 5.9 × 10^-19^ |
| *Vagococcus* | INI vs UNI75 | 1.56 × 10^-18^ | 2.08 × 10^-18^ |
| *Veillonella* | INI vs UNI0 | 9.85 × 10^-21^ | 3.94 × 10^-20^ |
| *Veillonella* | INI vs UNI25 | 3.34 × 10^-14^ | 3.34 × 10^-14^ |
| *Veillonella* | INI vs UNI50 | 2.95 × 10^-19^ | 5.9 × 10^-19^ |
| *Veillonella* | INI vs UNI75 | 1.56 × 10^-18^ | 2.08 × 10^-18^ |
| *Virgibacillus* | INI vs UNI0 | 9.85 × 10^-21^ | 3.94 × 10^-20^ |
| *Virgibacillus* | INI vs UNI25 | 3.34 × 10^-14^ | 3.34 × 10^-14^ |
| *Virgibacillus* | INI vs UNI50 | 2.95 × 10^-19^ | 5.9 × 10^-19^ |
| *Virgibacillus* | INI vs UNI75 | 1.56 × 10^-18^ | 2.08 × 10^-18^ |
| *Viridibacillus* | INI vs UNI0 | 9.85 × 10^-21^ | 3.94 × 10^-20^ |
| *Viridibacillus* | INI vs UNI25 | 3.34 × 10^-14^ | 3.34 × 10^-14^ |
| *Viridibacillus* | INI vs UNI50 | 2.95 × 10^-19^ | 5.9 × 10^-19^ |
| *Viridibacillus* | INI vs UNI75 | 1.56 × 10^-18^ | 2.08 × 10^-18^ |
| *W5053* | INI vs UNI0 | 9.85 × 10^-21^ | 3.94 × 10^-20^ |
| *W5053* | INI vs UNI25 | 3.34 × 10^-14^ | 3.34 × 10^-14^ |
| *W5053* | INI vs UNI50 | 2.95 × 10^-19^ | 5.9 × 10^-19^ |
| *W5053* | INI vs UNI75 | 1.56 × 10^-18^ | 2.08 × 10^-18^ |
| *Weissella* | INI vs UNI0 | 9.85 × 10^-21^ | 3.94 × 10^-20^ |
| *Weissella* | INI vs UNI25 | 3.34 × 10^-14^ | 3.34 × 10^-14^ |
| *Weissella* | INI vs UNI50 | 2.95 × 10^-19^ | 5.9 × 10^-19^ |
| *Weissella* | INI vs UNI75 | 1.56 × 10^-18^ | 2.08 × 10^-18^ |
