# Supplementary material for: Effects of Partial Fishmeal Replacement With Methanotrophic Bacterial Protein (Methylococcus capsulatus) in Siberian Sturgeon (Acipenser baerii) on Growth Performance, Nutrient Utilization, Body Composition, Immune Responses, Liver Histology, Gene Expression, and Gut Microbiota
Source: Aquac Nutr. 2026 Nov 2;2026:3788480. doi: 10.1155/anu/3788480 (PMC13728454; doi:10.1155/anu/3788480)
Supplement: Supplementary file 2 [file ANU-2026-3788480-s002.docx]

**List of supplemental figures**

**Fig. S1** Liver of Siberian sturgeon (*Acipenser baerii*) fed with dietary MCP, H&E, 400x. A – UNI0; B – UNI25; C – UNI50; D – UNI75. The arrowheads indicate vessels; the arrows mark exemplary lipidic hepatocytes; the asterisk mark extensive hyperaemic place.

**Fig. S2** Alpha diversity measured by Shannon and Simpson indexes. Data are shown as box plots (n = 9). The horizontal line indicates the median, and the whiskers indicate the lowest and highest points within the lower or upper quartile interquartile ranges.
